# A Systematic Review of Salt Reduction Initiatives Around the World: A Midterm Evaluation of Progress Towards the 2025 Global Non-Communicable Diseases Salt Reduction Target

**DOI:** 10.1093/advances/nmab008

**Published:** 2021-03-07

**Authors:** Joseph Alvin Santos, Dejen Tekle, Emalie Rosewarne, Nadia Flexner, Laura Cobb, Ayoub Al-Jawaldeh, Warrick Junsuk Kim, Joao Breda, Stephen Whiting, Norm Campbell, Bruce Neal, Jacqui Webster, Kathy Trieu

**Affiliations:** The George Institute for Global Health, University of New South Wales, Newtown, NSW, Australia; The George Institute for Global Health, University of New South Wales, Newtown, NSW, Australia; The George Institute for Global Health, University of New South Wales, Newtown, NSW, Australia; University of Toronto, Toronto, Ontario, Canada; Pan American Health Organization—World Health Organization Regional Office for the Americas, Washington, DC, USA; Resolve to Save Lives, An Initiative of Vital Strategies, New York City, NY, USA; World Health Organization Regional Office for the Eastern Mediterranean, Cairo, Egypt; World Health Organization Regional Office for the Western Pacific, Manila, Philippines; World Health Organization Regional Office for Europe, Copenhagen, Denmark; World Health Organization Regional Office for Europe, Copenhagen, Denmark; University of Calgary, Alberta, Canada; The George Institute for Global Health, University of New South Wales, Newtown, NSW, Australia; The George Institute for Global Health, University of New South Wales, Newtown, NSW, Australia; The George Institute for Global Health, University of New South Wales, Newtown, NSW, Australia

**Keywords:** salt intake, salt reduction, salt, sodium, food policy, public health nutrition, noncommunicable diseases

## Abstract

In 2013, the WHO recommended that all member states aim to reduce population salt intake by 30% by 2025. The year 2019 represents the midpoint, making it a critical time to assess countries’ progress towards this target. This review aims to identify all national salt reduction initiatives around the world in 2019, and to quantify countries’ progress in achieving the salt reduction target. Relevant data were identified through searches of peer-reviewed and gray literature, supplemented with responses from prefilled country questionnaires sent to known country leads of salt reduction or salt champions, WHO regional representatives, and international experts to request further information. Core characteristics of each country's strategy, including evaluations of program impact, were extracted and summarized. A total of 96 national salt reduction initiatives were identified, representing a 28% increase in the number reported in 2014. About 90% of the initiatives were multifaceted in approach, and 60% had a regulatory component. Approaches include interventions in settings (*n*= 74), food reformulation (*n *= 68), consumer education (*n *= 50), front-of-pack labeling (*n *= 48), and salt taxation (*n *= 5). Since 2014, there has been an increase in the number of countries implementing each of the approaches, except consumer education. Data on program impact were limited. There were 3 countries that reported a substantial decrease (>2 g/day), 9 that reported a moderate decrease (1–2 g/day), and 5 that reported a slight decrease (<1 g/day) in the mean salt intake over time, but none have yet met the targeted 30% relative reduction in salt intake from baseline. In summary, there has been an increase in the number of salt reduction initiatives around the world since 2014. More countries are now opting for structural or regulatory approaches. However, efforts must be urgently accelerated and replicated in other countries and more rigorous monitoring and evaluation of strategies is needed to achieve the salt reduction target.

## Introduction

Noncommunicable diseases (NCDs) are the leading cause of death worldwide, responsible for 41.5 million deaths in 2016 (71% of the 57 million deaths), more than all the other causes combined (1). Of the NCDs, cardiovascular diseases (CVDs) account for most deaths (1), and raised blood pressure is the leading risk factor for CVDs. The recent Global Burden of Disease study showed that high blood pressure accounted for 10.4 million deaths and 218 million disability-adjusted life years (DALYs) in 2017 (2), and excess salt intake—a well-established cause of high blood pressure (3–7)—was responsible for 3.2 million deaths and 70 million DALYs (2).

In 2013, the WHO made a recommendation to all Member States to reduce population salt intake by 30%, as part of the 9 global targets to reduce premature mortality from NCDs by 25% by 2025 (8). Reducing salt intake in populations is considered to be among the most cost-effective interventions to reduce the burden of NCDs (9, 10), and is therefore considered a priority action for all countries. In 2014, a systematic review of salt reduction initiatives around the world (11) reported that a total of 75 countries had national salt reduction initiatives in place, twice the number reported in 2010 (12). The approaches comprised food reformulation to reduce the salt content of products, consumer education, front-of-pack labeling schemes, salt taxation, and interventions in settings (11). A subsequent review reported the effects of these national-level initiatives in reducing salt consumption (13). In recognition that not all salt reduction interventions are conducted at the national level, another review of state- and community-level initiatives demonstrated that these actions were also effective (14), although it is notable that most of the interventions were carried out in high-income countries. In 2016, the WHO published the SHAKE Technical Package for Salt Reduction, to further support member states in carrying out salt reduction strategies through 5 key action areas: surveillance, harnessing industry, adopting standards for labeling, knowledge, and environment (15). This was published with the view that countries would adapt, implement, and translate this knowledge into effective policies and interventions. Continuous monitoring and evaluations of salt reduction initiatives are essential for knowledge sharing and identifying areas that require support.

The objective of this review is to identify national salt reduction initiatives around the world in 2019, with a view to quantify and describe countries’ progress in the implementation of salt reduction initiatives since the last review in 2014. This is critical, as 2019 is the midway point between the year the global salt target was endorsed (2013) and the year it should be achieved (2025).

## Methods

The systematic review protocol was registered at the International Prospective Register of Systematic Reviews as CRD42019133145. The study was approved by the University of New South Wales’ Human Research Ethics Committee (HC190243).

### Identification of countries with national salt reduction strategies

#### Search strategy

The search strategy employed in the previous review was followed (11). Briefly, national salt reduction initiatives were identified through a series of steps designed to maximize coverage. First, a search for published literature was conducted using MEDLINE, EMBASE, the Cochrane Central Register of Controlled Trials, the Cochrane Database of Systematic Reviews, the Cochrane Public Health Group Specialized Register, the Trials Register of Promoting Health Interventions, the Effective Public Health Practice Project, Web of Science, and the Latin American and Caribbean Health Sciences Literature database between January 2014 and December 2019. The search comprised 2 groups of terms: *1*) dietary salt or sodium; and *2*) salt reduction implementation strategies, including food reformulation, salt taxes, nutrition policies and interventions in settings, front-of-pack food labeling, and consumer education. Second, a search for gray literature was carried out using Open Grey, Google Scholar, and institutional websites, including the WHO regional office databases, WHO NCD document repository, Caribbean Food and Nutrition Institute, Center for Science in the Public Interest, World Action on Salt and Health, World Cancer Research Fund NOURISHING database, CDC, Institute of Medicine, and Public Health Agency of Canada resources. **Supplemental Table 1** lists the full search strategy used in MEDLINE, which was adapted to take into account differences in syntax rules across databases. The search was not restricted by language or study type.

#### Study selection and data extraction

Articles identified from the online searches were exported to EndNote X9 (Clarivate Analytics). Two authors (JAS and DT) independently screened the titles, abstracts, and the full texts of potentially relevant articles based on the inclusion and exclusion criteria detailed below. Disagreements at any stage of the screening process were resolved through consultation with a third review author (KT). Data extraction was then carried out by 1 review author (JAS), and a second reviewer (KT) checked the data for accuracy. Inconsistencies were resolved through discussion until consensus was reached. Included studies were organized according to the country of study; for each country, standard information relating to the core characteristics of the national salt reduction initiatives was extracted, similar to the process used in previous reviews (11, 12). This included information regarding: *1*) leadership and strategic approach; *2*) baseline assessments and monitoring; *3*) implementation strategies; and *4*) evaluation of program impact. **Supplemental Material 1** provides more details on the data extracted from each study. The extracted information was used to update the Database of National Salt Reduction Initiatives, which was established during the 2014 review (11).

#### Verifying information from country or regional program leaders and global experts in salt reduction

The questionnaire used in the previous review to verify and supplement the details of a country's salt reduction initiative was refined and pilot tested with 4 countries (**Supplemental Material 2**). Questionnaires were prefilled with existing information from the database (i.e., information from the previous review and new data from the online searches) for each country. The questionnaire was sent to 70 known country leads of salt reduction initiatives from governments and nongovernmental organizations (NGO) or salt champions who were mainly involved in the development or implementation of the country's salt reduction initiative. They were asked to review and add further details to the questionnaire. Furthermore, global experts in salt reduction and regional program leaders (mainly WHO representatives) were contacted to identify any other countries with salt reduction initiatives not captured by the searches, and to pass on the questionnaire to their contacts to gather more information. Additional data acquired from the questionnaire responses were used to update the database.

### Inclusion and exclusion criteria

Articles of any type were considered if they provided information relevant to the development, implementation, progress, monitoring, or evaluation of salt reduction initiatives that were national in approach and aimed at reducing population salt intake. As with the previous review (11), a national salt reduction initiative was defined as having government involvement and at least 1 of the following components: *1*) a document or statement highlighting the country's plan of action to reduce the population-level salt intake; *2*) a program that involves work with the food industry to decrease salt levels in foods; *3*) a consumer education or awareness campaign to improve knowledge, attitudes, and behaviors (KAB) towards salt specifically; *4*) a salt-specific front-of-pack labeling scheme; *5*) a policy that involves taxing salt, high-salt foods, or unhealthy foods, where the definition of unhealthy includes high salt or sodium; or *6*) salt-related initiatives in settings (e.g., food procurement policies with a salt criteria, voluntary guidelines for reducing salt content for foods, education regarding salt, menu labeling) such as schools, hospitals, workplaces, government offices, and food chains or outlets. Studies were deemed ineligible if they only provided baseline assessments of salt intake, salt levels in foods, KAB, or sources of salt in the diet, or if the primary purpose was expanding the evidence base on salt but without a discussion about developing or implementing a salt reduction initiative. Countries that only implemented consumer awareness campaigns where salt reduction messages were part of broader nutrition or healthy-eating topics (i.e., not salt-specific) were not considered to have a national salt reduction initiative.

### Data analysis

The synthesis of findings based on all country information collected was divided into 2 stages. First, for countries identified as having a national salt reduction initiative in the previous review, the analysis concentrated on summarizing the evidence of implementation since, identifying changes in the strategy, monitoring the country's progress in salt reduction, and evaluating the impact of the program. Second, for newly identified countries, the analysis focused on examining the core characteristics of the initiative, including government involvement and leadership, baseline assessments that informed the strategy, implementation strategies, and evaluations of the impact. Further, the national salt reduction initiatives were classified as being either planned (the action plan is still being developed; the action plan has been developed but there was no evidence of program implementation; or all the implementation approaches are still in the planning phase) or implemented (with at least 1 type of strategy being implemented, regardless of whether the other implementation strategies are in the planning phase). Data were also analyzed by the country's region based on the WHO regional classification (16) and by the country's income level based on the 2019 World Bank classification (17).

## Results

### Search results and sources of information

**FIGURE 1:**
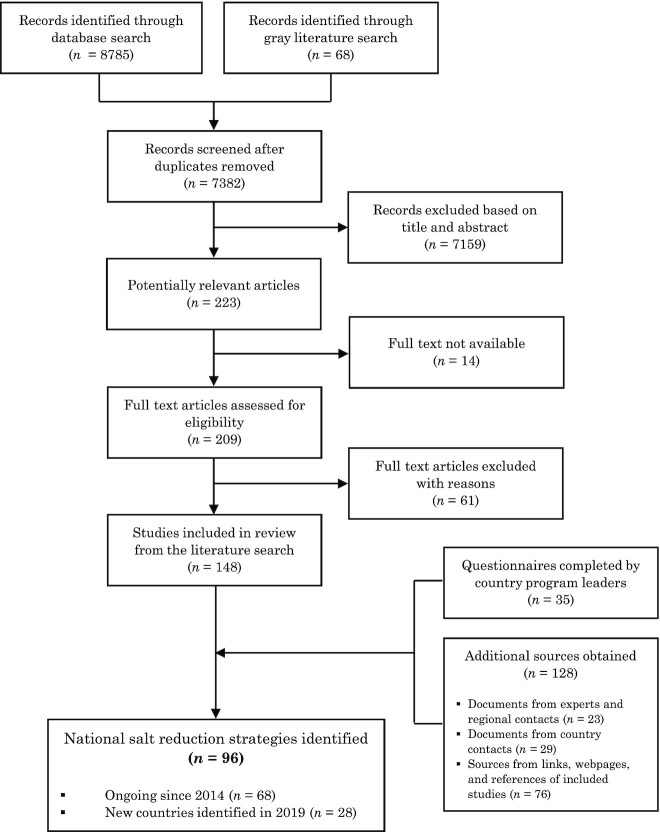
Identification of national salt reduction strategies around the world.

The initial peer-reviewed and gray literature search identified 148 articles with relevant information. An additional 128 documents or web pages were obtained through contact with salt reduction program leaders, WHO regional representatives, or international experts in salt reduction, and by review of reference lists of included studies. Furthermore, out of the 70 country questionnaires sent to known country contacts, 35 returned the questionnaire (50% response rate). Overall, in 2019 there were 96 national salt reduction initiatives identified (68 ongoing since 2014 and 28 newly identified;   **Figure 1**), with an additional 16 countries in their planning stages.

Of the 75 countries with national salt reduction initiatives in 2014, evidence of ongoing implementation was uncertain for 7 countries (i.e., no information was found from the search or through contact with stakeholders). Of the remaining 68 countries, information about progress or changes to the strategies since 2014 was confirmed for 30 through returned questionnaires; for other countries, information was obtained from the documents or webpages retrieved. In addition, unless there was any evidence of change or discontinuation, countries with initiatives that incorporated mandatory regulations or structural approaches, such as targets for salt levels in foods (mandatory or voluntary), front-of-pack labeling (mandatory or voluntary), salt taxation, and procurement policies or voluntary guidelines in settings were assumed to have continued with these approaches.

Of the 28 new countries in the implementation phase, 3 were verified through completed questionnaires, while the rest was based on information from the search or communication with stakeholders. Lastly, information on 16 countries currently in the planning phase to implement a salt reduction initiative was obtained from the search and documents provided by stakeholders, with 2 confirmed through returned questionnaires.

### Characteristics of national salt reduction initiatives

**FIGURE 2:**
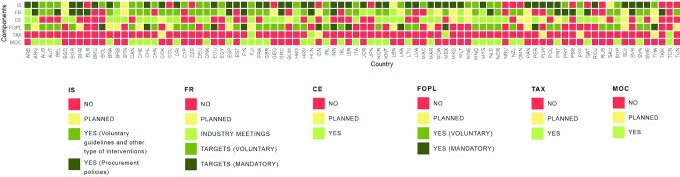
Characteristics of national salt reduction initiatives around the world (*n* = 96). For IS, some countries implemented multiple initiatives in different settings. If at least 1 initiative is a procurement policy, then IS was marked as “YES (procurement policies)” in this figure. The same rule applies to FR and FOPL (if at least 1 initiative is mandatory). MOC refers to changes in either salt intake, salt levels in foods, or KAB towards salt, that were measured using the same methods (i.e., comparable methods of assessment over time). The specific activities for each implementation strategy per country, the details on the measures of change, and the sources of information are shown in Supplemental Table 2. Abbreviations: CE, consumer education; FOPL, front-of-pack labeling; FR, food reformulation; IS, interventions in settings; KAB, knowledge, attitudes, and behaviors; MOC, measure of change; TAX, salt taxation.

Of the 96 national salt reduction initiatives, 89 (93%) were multifaceted in approach, characterized by a combination of 2 or more implementation strategies (**Figure 2**). Interventions in settings were the most common approach, with 74 countries (77%) implementing this type of strategy. This was followed by food reformulation through engagement with the food industry (68/96; 71%), consumer education interventions (50/96; 52%), front-of-pack labeling schemes (48/96; 50%), and salt taxation (5/96; 5%). **Supplemental Table 2** shows the detailed activities for each implementation strategy of the 96 national salt reduction initiatives.

- *Interventions in settings*. There were 74 countries with existing initiatives that target salt reduction in settings, while a further 6 countries were in the planning phase. Most countries (50/74) were implementing a mix of interventions in multiple settings. The majority of interventions were targeted at schools (70/74), followed by workplaces (25/74), fast-food chains or restaurants (21/74), hospitals (19/74), government offices (14/74), and other settings (9/74). Healthy food procurement policies (44/74), voluntary nutrition guidelines (29/74), and education (25/74) were the most common interventions in these settings.
- *Food reformulation*. In all, 68 countries were working with the food industry: 11 countries had meetings and/or voluntary agreements to lower salt levels in foods, and 57 countries had taken the next step and established salt targets. Thirteen countries were planning to implement food reformulation strategies. Of the 57 countries with salt targets, 19 have mandatory maximum salt limits for foods. Half of these countries set mandatory targets solely for bread (Bahrain, Belgium, Hungary, Netherlands, Palestine, Paraguay, Portugal, Qatar, Spain, and Turkmenistan), while the other half covered a wider range of foods, including processed meats, cheeses, crisps and snacks, soups and stocks, canned fish, tomato products, and fruit and vegetables (Argentina, Belarus, Bulgaria, Finland, Greece, Iran, Slovakia, South Africa, and Uzbekistan). There were 48 countries with voluntary salt targets in place, with a range in the number of food categories and type of products (within categories) being targeted for reformulation. Finally, 10 countries have a mix of mandatory and voluntary salt targets in their food reformulation scheme.
- *Consumer education*. While most countries implemented consumer awareness campaigns in relation to overall nutrition and health, 61 countries have initiated (*n* = 50) or planned (*n* = 11) consumer education programs that include a salt reduction component since 2014. All but 1 country were implementing or planning to implement this intervention in conjunction with other strategies. Of the 50 existing consumer education programs, 38 were led solely by the government, 3 led solely by an NGO, and 9 by shared leadership between the government, an NGO, or the food industry.
- *Front-of-pack labeling*. There were 48 countries with a front-of-pack labeling scheme that accounts for sodium or salt content, while an additional 16 countries were in the planning phase. A mandatory approach has been adopted in 12 countries through warning labels (6/12), traffic lights (4/12), health messages (3/12), and percentage daily intake or guideline daily amount (2/12). In addition, 41 countries have voluntary schemes (5 countries have both mandatory and voluntary schemes in place). These included endorsement logos and symbols (19/41), percentage daily intake or guideline daily amount (15/41), traffic lights (7/41), ratings (3/41), and health messages (2/41). Examples of endorsement logos and symbols used in different countries were the healthy choices logo (Belgium, Czechia, Malaysia, Poland, and Thailand), the keyhole logo (Denmark, Iceland, Lithuania, Norway, and Sweden), the heart symbol (Finland), the healthy living guarantee mark (Croatia), and the protective food logo (Slovenia). Rating systems such as the Health Star Rating and Nutri-score were being used in Australia and New Zealand, and France respectively.
- *Salt taxation*. There were 5 countries—Fiji, Hungary, Mexico, Saint Vincent and the Grenadines, and Tonga—that adopted taxes on foods high in salt. In these countries, the tax on high-salt foods is being implemented as part of a broader set of taxes that cover other nutrients or foods. In Fiji, import taxes were applied to monosodium glutamate and palm oil. In Hungary, a public health product tax is in place, encompassing a variety of prepackaged foods, including salty snacks and condiments that exceed recommended salt limits. In Mexico, an 8% tax applies to nonessential foods that exceed energy density limits, including salty snacks. In Saint Vincent and the Grenadines, a 15% value-added tax was placed on salt, sugar, and sweetened beverages in 2016. In Tonga, an excise tax on unhealthy foods, including turkey tails, mutton flaps, chicken legs, and other high-salt foods, such as instant noodles, was introduced in 2015. An additional 5 countries (Cook Islands, French Polynesia, Indonesia, Palau, and Thailand) were considering salt taxation. In Thailand, the government proposed a salt tax (2019) that will target salty foods such as frozen products, canned foods, and instant noodles. In Portugal, a proposed tax on foods high in salt was considered in 2018 but ultimately not approved by the Parliament.

### Implementation progress since 2014

**FIGURE 3:**
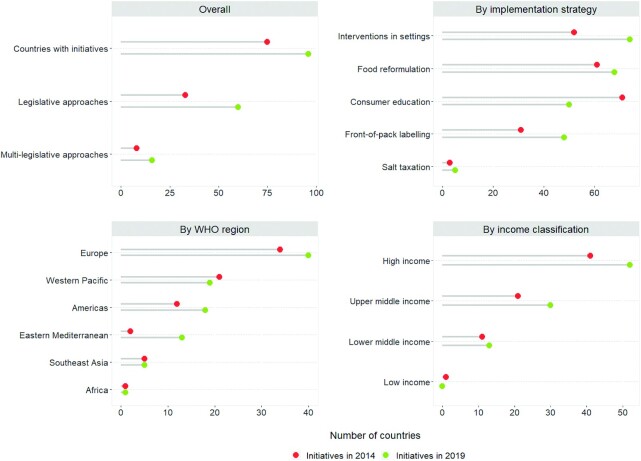
Summary of progress in implementing salt reduction initiatives since 2014.

Overall, there has been a 28% increase in the number of national salt reduction initiatives, from 75 in 2014 to 96 in 2019. In terms of implementation strategy, there have been increases in the number of countries implementing interventions in settings (from 52 in 2014 to 74 in 2019), food reformulation approaches (from 61 to 68), front-of-pack labeling schemes (from 31 to 48), and salt taxation (from 3 to 5) (**Figure 3**). More specifically, more countries are now implementing mandatory targets (from 9 to 19) or voluntary (from 36 to 48) targets for salt content in foods, mandatory (from 8 to 12) or voluntary (from 26 to 41) front-of-pack labeling schemes, and procurement policies (from 23 to 44) or voluntary nutrition guidelines (from 19 to 29) in settings. In contrast, the number of countries reporting consumer education programs fell from 71 in 2014 to 50 in 2019.

### National salt reduction initiatives by WHO region

Of the 96 national salt reduction initiatives identified, 40 were in Europe, 19 in the Western Pacific, 18 in the Americas, 13 in the Eastern Mediterranean, 5 in Southeast Asia, and 1 in Africa. Since 2014, there have been increases in the number of initiatives in Europe (by 6), the Americas (by 6), and the Eastern Mediterranean (by 11); no changes in Africa and Southeast Asia; and there has been a decrease in the number of initiatives in the Western Pacific (by 2; Figure 3).

**FIGURE 4:**
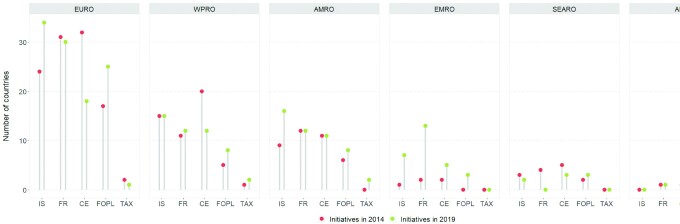
Summary of progress in implementing salt reduction initiatives by WHO region. Abbreviations: AFRO, Regional Office for Africa; AMRO, Regional Office for the Americas; CE, consumer education; EMRO, Regional Office for the Eastern Mediterranean; EURO, Regional Office for Europe; FOPL, front-of-pack labeling; FR, food reformulation; IS, intervention in settings; SEARO, Regional Office for Southeast Asia; TAX, salt taxation; WPRO, Regional Office for the Western Pacific.

- *Europe*. In this region, 40/53 (75%) countries/territories have existing national salt reduction initiatives. An additional 5 countries were in the planning phase, including Luxembourg, which was identified as implementing an initiative in 2014, but no evidence of continuing implementation was found since. However, the country's recent National Nutrition Plan (2018–2025) includes a proposal to create a working group to develop strategies to reduce salt levels in foods. There were 8 countries with no identified initiative. Interventions in settings (34/40) and food reformulation (30/40) were the most commonly used approaches, followed by front-of-pack labeling (25/40; **Figure 4**). Across all regions, Europe has the highest number of countries implementing mandatory targets for salt levels in foods (*n* = 12).
- *Western Pacific*. In this region, 19/37 (51%) countries/territories/areas have national salt reduction initiatives in place, including 3 new initiatives (Guam, Hong Kong, and Macao) since the 2014 review; there is also 1 initiative in the planning phase (Vanuatu). There were 12 countries/territories/areas with no identified initiative. There were 5 Pacific Island countries (Federated States of Micronesia, Marshall Islands, New Caledonia, Solomon Islands, and Tuvalu) identified to be conducting industry meetings and/or consumer awareness campaigns in 2014, but we found no evidence of continuing implementation of these activities since; therefore, these countries were not counted, which explains the decline in the number of identified initiatives in this region. Interventions in settings (15/19), food reformulation through industry meetings and voluntary targets for salt levels in foods (12/19), and consumer education programs (12/19) were the main approaches, followed by voluntary front-of-pack labeling (8/19).
- *Americas*. In the Americas, 18/49 (37%) countries/territories have a national salt reduction initiative, with a further 6 countries in the planning stage, and 24 without an initiative. One country (Cuba) was previously identified in 2014, but we found no evidence of implementation since. The main implementation approaches [interventions in settings (16/18); food reformulation approaches (12/18); consumer education (11/18); and front-of-pack labeling (8/18)] were similar to those in the other regions. Of all the regions, the Americas has the highest number of mandatory front-of-pack labeling schemes (*n* = 6).
- *Eastern Mediterranean*. From only 2 countries identified in the previous review, this region now has 13/22 (59%) countries with a national salt reduction initiative. All 13 countries work with the food industry to reduce salt levels in foods, through meetings or agreements (5/13), voluntary targets for food categories (4/13), and/or mandatory targets (4/13). In this region, 5 countries have implemented consumer education programs, 7 countries carry out work in settings, and 3 countries have a front-of-pack labeling scheme.
- *Southeast Asia*. As in 2014, 5/11 (45%) countries have a national salt reduction initiative in this region, 2 are in the planning stage and 4 are without an initiative. There are 3 countries implementing consumer education programs and front-of-pack labeling schemes, while all 5 with initiatives are in the planning phase to carry out food reformulation strategies.
- *Africa*. The only country with a national salt reduction initiative in this region is South Africa (1/47; 2%), although 2 countries (Ethiopia and Nigeria) are in the planning phase. South Africa has mandatory targets for salt levels in foods, a voluntary front-of-pack labeling scheme, and consumer education programs.

### National salt reduction initiatives by income classification

**FIGURE 5:**
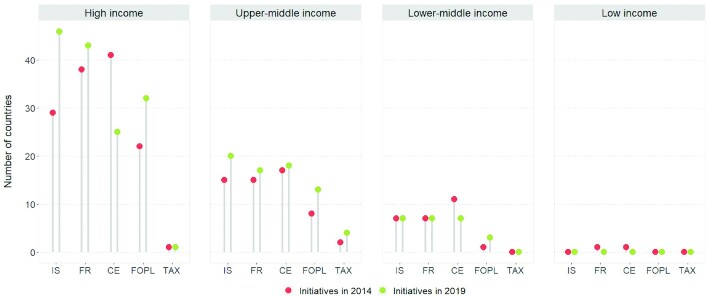
Summary of progress in implementing salt reduction initiatives by income classification. Abbreviations: CE, consumer education; FOPL, front-of-pack labeling; FR, food reformulation; IS, intervention in settings; TAX, salt taxation.

Using the World Bank's 2019 income classification, national salt reduction initiatives are now in place in 52 high-income countries (from 41 in 2014), 30 upper-middle–income countries (from 21), 13 lower-middle–income countries (from 11), and 1 country that is unclassified. Apart from salt taxation, which is missing from lower-middle–income countries, all forms of implementation strategies are present in the 3 income groups. Interventions in settings (46/52), food reformulation (43/52), and front-of-pack labeling (32/52) were the most common strategic approaches in high-income countries, while interventions in settings (20/30 and 7/13, respectively), food reformulation (17/30 and 7/13, respectively), and consumer education (18/30 and 7/13, respectively) were common in upper-middle– and lower-middle–income countries. In terms of food reformulation strategies, 37 high-income, 14 upper-middle–income, and 5 lower-middle–income countries have existing mandatory or voluntary targets for salt levels in foods. Finally, of the 5 countries that have adopted taxation on high-salt foods, 1 is high-income and 4 are upper-middle–income countries. No initiative has been identified in low-income countries. It is to note that the only low-income country (Bangladesh) identified in the previous review as having an initiative has been reclassified as a lower-middle–income country in the present review, based on the World Bank's 2019 income classification. **Figure 5** displays the changes in the implementation strategies in the income groups since 2014.

### Reported data on changes in salt intake, salt levels in foods, and KAB towards salt

Since 2014, there have been increases in the number of countries with measures of change in salt intake (25/96, from 12/75 in 2014), change in salt content of foods (31/96, from 19/75), and change in KAB towards salt (19/96, from 7/75). In terms of change in salt intake, 11 countries reported changes using 24-hour urine samples; 10 using dietary-based assessment methods, such as 24-hour dietary recalls and food records; 2 based on total diet studies; 1 using spot urine samples; and 1 using unspecified methods (**Table 1**). Based on these data, 3 countries reported a substantial decrease (>2 g/day), 9 a moderate decrease (1–2 g/day), and 5 a slight decrease (<1 g/day) in mean salt intake over time, while 1 country had a slight increase in salt intake. There were 7 countries with minimal to no change (<0.5 g/day) in salt intake between measurements.

**TABLE 1:** Countries with reported data on change in mean salt intake over time

Country (Reference)	Timescale	Assessment method	Change in salt intake, g; percent change from baseline
Argentina (18)	2011–2015	24-hour urine	11.2g to 9.2g; −18%
Australia^1^ (19)	2011–2014	24-hour urine	*Victoria*
			7.9g to 7.8g; −1%
Austria (20)	2008–2012	24-hour diet recall	8.3g to 8.2g; −1%
Canada (21)	2004–2015	24-hour diet recall	8.5g to 6.9g; −19%
China (22)	2000–2009/12	Total diet study	11.8g to 9.1g; −23%
Denmark (11)	2006–2010	Spot urine	M: 10.7g to 9.9g; −7%
			F: 7.5g to 7.0g; −7%
Fiji (23)	2012/13–2015/16	24-hour urine	11.7g to 10.3g; −12%
Finland^1^ (20, 24)	1979–1987	24-hour urine	*Kuopio area*
			M: 13.1g to 12.0g; −8%
			F: 10.4g to 9.5g; −9%
	1979–2002		*North Karelia*
			M: 12.9g to 9.5g; −26%
			F: 10.4g to 7.4g; −29%
	1982–2002		*Southwestern Finland*
			M: 11.6g to 9.8g; −16%
			F: 9.1g to 7.4g; −19%
France (20)	1998/99–2015/16	7-day food record	8.0g to 7.5g; −6%
Iceland (11, 25)	2002–2010/11	24-hour diet recall	8.4g to 7.9g; −6%
Ireland (11)	2001–2011	4-day food record	8.1g to 7.0g; −14%
Italy (26)	2009/10–2012	24-hour urine	M: 10.6g to 9.3g; −12%
			F: 8.3g to 7.2g; −13%
Japan (27)	2011–2017	Dietary record	9.9g to 9.4g; −5%
Lithuania (11, 28)	2007–2013/14	24-hour diet recall	8.8g to 7.1g; −19%
Netherlands (29)	2006–2015	24-hour urine	M: 9.9g to 9.7g; −2%
			F: 7.9g to 7.4g; −6%
New Zealand (30)	2009–2016	Total diet study	M: 7.3g to 7.2g; −1%
			F: 5.1g to 5.2g; +2%
Portugal (31)	Unknown	Unknown	−1.7g reduction
Samoa (32)	2013–2015	24-hour urine	7.3g to 7.5g; +3%
Singapore (Q2019)	1998–2018	Dietary survey	9.0g to 9.0g; 0%
Slovenia (33)	2007–2012	24-hour urine	M: 14.3g to 12.9g; −10%
			F: 11.0g to 10.7g; −3%
South Korea (34)	2005–2015	Dietary survey	13.2g to 9.7g; −27%
Switzerland (20)	1984–2011	24-hour urine	8.4g to 9.2g; +10%
Turkey (35)	2008–2012	24-hour urine	18.0g to 15.0g; −17%
United Kingdom (36)	2005/6–2018/19	24-hour urine	8.1g to 7.5g; −7%
United States (37, 38)	2011/12–2015/16	24-hour diet recall	8.7g to 8.5g; −2%

Includes data on changes in salt intake measured using comparable assessment methods over time. Abbreviations: F, female; M, male; Q2019, information obtained from country questionnaire 2019.

1 Change in salt intake measured at the subnational level.

In terms of change in salt levels in foods, the methods used to evaluate changes also varied, with 9 countries using food analyses, 6 using food label surveys, 3 using industry self-report data, 1 using existing databases, 1 using household consumer panel or sales data, and 6 applying a combination of these approaches. The types of foods covered in the evaluations varied in scope, with some countries covering over 50 food categories while some assessing a single category. Bread was the most commonly targeted product, although it is notable that the definitions and products included as “bread” differed across the countries. Overall, the majority of countries reported reductions in salt content of bread; however, countries that evaluated multiple food categories also showed increases in salt content in some foods, and no to minimal changes in others. Lastly, the change in KAB in relation to salt were based on surveys/self-reported questionnaires. The studies measured different aspects of KAB (so it was not easy to compare data between studies), but most countries showed improvements in either knowledge, attitudes, or behaviors towards salt intake. Supplemental Table 2 provides more details about the data collection period and the methods of assessment of change in salt levels in foods and KAB towards salt.

## Discussion

This review demonstrated that there has been an increase in the number of countries with national salt reduction initiatives from 2014 to 2019. While the relative increase from 2010 to 2014 (134% increase) shown in the previous review (11) was considerably larger compared to what was recorded in our current review (28% increase from 2014 to 2019), the existing national salt reduction initiatives in 2019 (*n* = 96) represent about 50% of all the Member States of the United Nations. However, despite the increase in the number of countries adopting strategies to reduce their population-level salt intake, more action is needed to ensure that countries monitor and evaluate their strategies, and accelerate their efforts to achieve the targeted 30% reduction in salt intake. While it is encouraging to see that more countries are now reporting data on changes in salt intake (25/96), it is important to note that some of the reported data were at the subnational level and some were outdated, while less than half of the countries employed the gold standard 24-hour urine collection. Some countries have data available over multiple periods but the methods of assessment were not comparable between time points; hence, these data were excluded from our analyses. While 17 countries have demonstrated a slight (<1 g/day) to substantial (>2 g/day) decrease in salt intake over time, none have yet met the targeted 30% relative reduction in mean salt intake from baseline or achieved the recommended daily limit of 5 g/day. An in-depth evaluation of changes in salt intake as a result of population-level salt reduction interventions will be published in a separate review. More regular evaluations of national salt reduction initiatives are needed to understand what works; particularly, interim evaluations are needed during the life of the strategy (rather than just at the end of the intervention) so that necessary adaptations can be made to ensure the strategy effectively reduces salt intake (39, 40). As measuring changes in population salt intake regularly is complex and costly, process evaluations that examine the implementation progress, process indicators, and existing barriers and facilitators of implementation are likely more feasible and informative for identifying those areas requiring adaptation (40).

The main implementation strategies were interventions in settings, food reformulation, consumer education, front-of-pack labeling, and salt taxation. There has been an increase in the number of countries applying each of these approaches since 2014, apart from consumer education. The notable decline in consumer education programs (30% decrease) might be due in part to the lower rate of country questionnaire responses in the current review compared to the previous review (50% and 79%, respectively), given that much of the information about salt-specific consumer awareness campaigns was previously obtained from the questionnaire. Another possible reason might be because consumer education is usually done at the start of an intervention but may not be maintained, since it is resource- and time-intensive (41, 42). However, the increases in uptake for the other approaches suggest that more countries are now incorporating structural and policy-based initiatives—focusing on changing the food environment—to make it easier for consumers to make better food choices and eat less salt. In fact, in terms of regulatory approaches to salt reduction (i.e., mandatory targets for salt levels in foods, mandatory front-of-pack labeling, mandatory nutrition standards in settings, and salt taxation), the number of countries with such measures increased by 82% since 2014; also, 16 countries have more than 1 type of regulation aimed at lowering salt intake, compared to 8 countries in 2014. Furthermore, of the 28 newly identified national salt reduction initiatives, 61% have a regulatory component. Previous systematic reviews and modeling studies have shown that mandatory or legislative approaches may be more effective, and may produce larger reductions in population salt intake (43–45). Continuous monitoring and reporting of progress of each intervention approach is essential to assess whether these policies work, identify gaps, and understand how they can be better applied to lower salt intakes in populations with different contexts and dietary sources of salt.

As with the previous review, all WHO regions have national salt reduction initiatives in place. However, progress in terms of the number of initiatives varied markedly by region. The Eastern Mediterranean region recorded the largest increase since 2014 (from 2 to 13 countries), which reflects the concerted efforts led by the WHO Regional Office to provide a regional framework for action and to hold a series of meetings on reducing population salt intake (46). Similarly, there have been increases in the number of initiatives in the Americas and European regions, with over a third and 75% of countries in the regions, respectively, having a national salt reduction initiative. These 2 regions also have the highest numbers of countries that are currently in the planning stages (6 and 4, respectively). The increases in the number of initiatives in these 2 regions may be attributed to their ongoing regional collaboration, facilitated by the WHO European Salt Action Network, and the Pan American Health Organization and the Network of Action on Strategies for Reducing Sodium Consumption and Prevention and Control of Cardiovascular Disease in the Americas and the Caribbean (47, 48). In contrast, there have been no changes in the Southeast Asian and African regions, although this may be partially due to gaps in the published literature and fewer completed country questionnaires (*n* = 2). Lastly, in the Western Pacific region, there were 5 Pacific Island countries where we were unable to find evidence of continuing implementation since 2014. We speculate that this is due to the short-term nature of the implementation strategies (i.e., industry meetings and/or consumer awareness campaigns) identified in 2014 for these 5 countries, as well as a lack of information from the literature and the absence of completed country questionnaires to assess whether these strategies were continuing. Unlike structural or policy-based initiatives, we did not assume that these initiatives have continued since the last review. However, 3 new countries in the Western Pacific region were identified to have a salt reduction strategy in place, which suggests that salt-related activities are present in the region, although the extent of implementation appears to vary across countries. The establishment of regional networks that facilitate knowledge sharing, particularly among countries with similar sources of salt and dietary habits, may help increase adoption of national salt reduction initiatives.

There have been increases in the number of national salt reduction initiatives across income groups: by 27%, 43%, and 18% from high-income, upper-middle–income, and lower-middle–income countries, respectively. In both the high-income and upper-middle–income groups, more countries have incorporated some element of food reformulation, front-of-pack labeling schemes, and interventions in settings in their salt reduction initiative. With packaged or processed foods recognized as contributing about 75% of the daily salt intake in many high-income countries, and the increasing trend of processed food consumption in lower-middle–income and upper-middle–income countries (49, 50), these strategies might bring more positive changes in salt reduction in these contexts. However, in countries where salt mainly comes from discretionary sources added by the consumer during cooking or at the table—a trend that is common in many low- and middle-income countries—interventions that change consumer salt practices or replace salt with salt substitutes might be more relevant. Nevertheless, a multi-component approach in salt reduction—that considers structural or policy-based means together with consumer education—is recommended (43, 51, 52). Lastly, while we have seen positive developments in the other income groups, there has been a gap in the implementation of national salt reduction initiatives in low-income countries. Although the contribution of high-salt diet to cardiovascular disease burden is lowest in low-income countries compared to the other income groups (53), additional support is still warranted to develop policies and interventions to reduce excess salt intakes in low-income countries, particularly those experiencing a nutrition transition towards greater intake of processed and packaged foods.

This review has a number of strengths and limitations. The work reported here builds on the growing Database of National Salt Reduction Initiatives established in 2014, updated through our comprehensive data collection approach that involved a search of the peer-reviewed and gray literature, contact with country program leaders or salt champions, and coordination with regional WHO representatives and global experts in salt reduction. We triangulated multiple sources of data to understand the implementation of strategies, and the information obtained from all the sources was documented in a standardized manner. Through this, it is unlikely that any major national salt reduction initiatives have been missed, although we cannot exclude that possibility. The questionnaire from the 2014 review was updated and revised based on the lessons learned from the 2014 questionnaire, and was pilot tested in 4 countries. However, we obtained a lower response rate (50%) in the current review, so we were unable to verify some inconsistencies or obtain contemporary information about the state of salt reduction initiatives in some countries. In addition, our review did not include subnational salt reduction initiatives (apart from some interventions in public institution settings), which also contribute to lower salt intake. Risk of bias or quality assessments of the studies were deemed inapplicable given that multiple sources were used for each data point.

In conclusion, the review showed a further increase in the number of national salt reduction strategies around the world, to 96 countries, since 2014. More countries are now opting for structural or regulatory implementation strategies, such as targets for salt levels in foods, food procurement policies or nutrition standards in settings, front-of-pack labeling schemes, and salt taxation. The WHO regions of the Eastern Mediterranean, Europe, and the Americas demonstrated the largest increases in national salt reduction initiatives. Increased implementation of initiatives was noted in high-income and middle-income countries, but not in low-income countries. However, despite the increase in the number of countries adopting salt reduction strategies, none have yet met the targeted 30% relative reduction in salt intake, which was set to be achieved by 2025. Countries need to accelerate their efforts to achieve the targeted reduction, monitor and evaluate their strategies more regularly using reliable methods, and share lessons about the successful ingredients to their strategies to ensure countries collectively achieve the global salt reduction target by 2025.

## Supplementary Material

nmab008_Supplemental_File

## Notes

The authors reported no funding received for this study.

Author disclosures: JAS is supported by a National Health and Medical Research Council (NHMRC) Postgraduate scholarship (1168948). ER is supported by a University of New South Wales University Postgraduate Award (00889665). NC was a paid consultant to the Novartis Foundation (2016–2017) to support their program to improve hypertension control in low- to middle-income countries, which includes travel support for site visits and a contract to develop a survey; has provided paid consultative advice on accurate blood pressure assessment to Midway Corporation (2017); and is an unpaid member of World Action on Salt and Health. JW is the Director of the WHO Collaborating Centre on Population Salt Reduction; is supported by a National Heart Foundation Career Development Fellowship (1082924); and receives additional funding for work on salt reduction from NHRMC, the Victorian Health Promotion Foundation, Vital Strategies, and the WHO. KT is supported by an NHMRC Early Career Fellowship (1161597) and a Postdoctoral Fellowship (102140) from the National Heart Foundation of Australia. BN, JW, and KT are supported by NHMRC Centre of Research Excellence on food policy interventions to reduce salt (1117300). AA-J, WJK, JB, and SW are staff members of the WHO. DT, NF, and LC, no conflicts of interest.

The writing group takes sole responsibility for the content of this article, and the content of this article reflects the views of the authors only. The WHO is not liable for any use that may be made of the information contained therein.

Supplemental Tables 1 and 2 and Supplemental Material 1 and 2 are available from the “Supplementary data” link in the online posting of the article and from the same link in the online table of contents at https://academic.oup.com/advances/.

Abbreviations used: CVD, cardiovascular disease; DALY, disability-adjusted life year; KAB, knowledge, attitudes, and behaviors; NCD, noncommunicable disease; NGO, nongovernmental organization.
