# Supplementary material for: A Systematic Review of Salt Reduction Initiatives Around the World: A Midterm Evaluation of Progress Towards the 2025 Global Non-Communicable Diseases Salt Reduction Target
Source: Adv Nutr. 2021 Mar 7;12(5):1768–80. doi: 10.1093/advances/nmab008 (PMC8483946; doi:10.1093/advances/nmab008)
Supplement: nmab008_Supplemental_File [file nmab008_supplemental_file.zip › Supplementary_data_1.docx]

**Supplemental Table 1.** Full search strategy in MEDLINE

| **NO** | **SEARCHES** |
| --- | --- |
| 1 | sodium, dietary/ or sodium chloride, dietary/ |
| 2 | Sodium Chloride/ |
| 3 | Diet, Sodium-Restricted/ |
| 4 | ((salt or sodium) adj10 (reduc* or target* or cutback* or decreas* or limit* or consumption)).tw. |
| 5 | ((diet* or nutrition* or food or intake) adj10 (salt or sodium)).tw. |
| 6 | 1 or 2 or 3 or 4 or 5 |
| 7 | potassium.tw. |
| 8 | potassium/ |
| 9 | potassium, dietary/ |
| 10 | potassium chloride.tw. |
| 11 | potassium chloride/ |
| 12 | 7 or 8 or 9 or 10 or 11 |
| 13 | 6 or 12 |
| 14 | Food, Formulated/ |
| 15 | Food-Processing Industry/ |
| 16 | food technology/ or food analysis/ or food preservation/ |
| 17 | Food Industry/ |
| 18 | 15 or 16 or 17 |
| 19 | (adjust* or alter* or change or changing or control* or decreas* or limit* modify or modified or new or reduce or reducing or reduction* or reformulat* or redevelop* or restrict*).tw. |
| 20 | 18 and 19 |
| 21 | ((adjust* or alter* or change or changing or control* or decreas* or limit* or modify or modified or new or reduce or reducing or reduction* or reformulat* or redevelop* or restrict*) adj10 (recipe* or food or foods or formula* or ingredient*)).tw. |
| 22 | 14 or 20 or 21 |
| 23 | taxes/ or tax exemption/ |
| 24 | Government Programs/ |
| 25 | financing, organized/ or financing, government/ |
| 26 | "Cost Sharing"/ |
| 27 | (pricing or cost or costs or subsidi*).tw. |
| 28 | (taxation or taxes or subsid*).tw. |
| 29 | (financial adj3 (incentive* or disincentive*)).tw. |
| 30 | 23 or 24 or 25 or 26 or 27 or 28 or 29 |
| 31 | Nutrition Policy/ |
| 32 | exp Food Service, Hospital/ |
| 33 | Food Services/ |
| 34 | schools/ or schools, nursery/ |
| 35 | Workplace/ |
| 36 | Prisons/ |
| 37 | Universities/ |
| 38 | Child Day Care Centers/ |
| 39 | ((food* or menu or nutrition*) adj5 (buy* or procur* or purchas* or stock*) adj5 (guideline* or policy or policies or practice* or standard*)).tw. |
| 40 | ((cafeteria* or diet or food* or menu* or nutrition*) adj10 (childcare or child-care or college* or daycare* or day-care* or fitness centre* or fitness center* or hospital* or leisure center* or leisure centre* or preschool* or pre-school* or prison* or public facilit* or recreation center* or recreation centre* or recreation facilit* or school* or universit*)).tw. |
| 41 | 31 or 32 or 33 or 34 or 35 or 36 or 37 or 38 or 39 or 40 |
| 42 | Advertising as Topic/ |
| 43 | ((market* or adverti* or promot*) adj10 (adolescent* or adolescence or child or children or teenager* or teens or young people or youth*)).tw. |
| 44 | 42 or 43 |
| 45 | Food Labeling/ |
| 46 | Food Packaging/lj, st [Legislation & Jurisprudence, Standards] |
| 47 | ((food* or nutrition* or diet*) adj10 (facts or information or label* or symbol* or warning*)).tw. |
| 48 | health check.tw. |
| 49 | 45 or 46 or 47 or 48 |
| 50 | nutrition surveys/ or diet surveys/ |
| 51 | communications media/ or exp mass media/ |
| 52 | Social Marketing/ |
| 53 | health education/ or exp consumer health information/ or health fairs/ |
| 54 | exp Health Promotion/ |
| 55 | Information Dissemination/ |
| 56 | newspapers/ or periodicals as topic/ |
| 57 | computer communication networks/ or internet/ or blogging/ or social media/ |
| 58 | Electronic Mail/ |
| 59 | ((communicat* adj2 campaign*) or (information adj2 campaign*) or mass media or newspaper* or television* or radio* or (public adj2 campaign*) or (national adj2 campaign*) or public information).tw. |
| 60 | (blog* or email* or facebook or internet or magazine* or mobile device* or PDA or SMS or smartphone* or social media or text messag* or twitter or web).tw. |
| 61 | (health education or health information or health promotion).tw. |
| 62 | 50 or 51 or 52 or 53 or 54 or 55 or 56 or 57 or 58 or 59 or 60 or 61 |
| 63 | ("salt substitution" or "salt substitute" or "low-sodium salt substitute" or "salt replacing" or "salt replacement" or "salt replacer" or "salt reduction" or "salt reducer" or "Low-So salt replacer" or "KcLean salt" or "Kalisel" or "Salt Trim" or "Lacto Optitaste" or "Pansalt" or "Sub4salt" or "LomaSalt" or "Saltwise" or "Myciscent" or "Salt reducer N100" or "Salt reducer N200" or "Dr Lohmann’s Premix salt replacer" or "AlsoSalt" or "Nu-Tek’s modified potassium chloride" or "Soda-Lo" or "Zalt" or "Maxorite delite" or "Maxarite Bsalt" or "Maxarite Dsalt" or "Maxarome select" or "Maxarome pure" or "KojiAji" or "Ajimate super RK" or "Ajinomoto" or "SaltAnswer" or "Super YE" or "Fonterra Savoury Powder" or "Flavour intensifier" or "Savoury Flavour enhancer" or "Flavour enhancer" or "SavourCrave" or "UnSal20" or "Seagreens Organic Mineral Salt" or "Sense Capture Salt" or "magnesium" or "MgCl").mp. |
| 64 | 22 or 30 or 41 or 44 or 49 or 62 or 63 |
| 65 | 13 and 64 |
| 66 | exp animals/ not humans.sh. |
| 67 | 65 not 66 |
| 68 | limit 67 to yr="2014 -Current" |
