# Supplementary material for: A Systematic Review of Salt Reduction Initiatives Around the World: A Midterm Evaluation of Progress Towards the 2025 Global Non-Communicable Diseases Salt Reduction Target
Source: Adv Nutr. 2021 Mar 7;12(5):1768–80. doi: 10.1093/advances/nmab008 (PMC8483946; doi:10.1093/advances/nmab008)
Supplement: nmab008_Supplemental_File [file nmab008_supplemental_file.zip › Supplementary_data_2.docx]

**Supplemental Material 1.** Characteristics of salt reduction strategies extracted

*From Trieu et al, 2015. Salt reduction initiatives around the world – A systematic review of progress towards the global target. PLoS ONE 10(7): e0130247.*

**Leadership and strategic approach**

1. Leadership: the organization leading or coordinating the programme
2. Dietary targets: presence of an agreed national population target for salt consumption with target amount
3. Programme specificity: whether the strategy was salt-specific or a part of a broader health programme
4. NGO/advocacy action: the presence of consumer/advocacy organizations working on salt

**Baseline assessments and monitoring**

1. Salt intakes: estimated mean baseline salt intakes and method of measurement
2. Salt levels in foods: whether countries had recorded salt levels in processed foods and method of measurement
3. Consumer awareness: whether countries had a baseline measure of consumer awareness or behaviours
4. Monitoring: whether or not monitoring systems are in place in relation to each of these criteria

**Implementation strategies**

1. Food reformulation: whether the strategy included engagement with the food industry to reformulate foods and whether the approach was voluntary or mandatory
2. Targets for salt levels in foods: whether the program of work with the food industry included voluntary or mandatory salt level targets for foods
3. Consumer behaviour: whether there was a salt-specific consumer awareness campaign and whether this was led by a government or NGO or other organisations
4. Labelling: whether front-of-pack labelling schemes such as warnings, traffic lights, percentage daily intake or guideline daily amount, or symbol or logo scheme had been introduced as part of the salt reduction programme and whether this was voluntary or mandatory
5. Taxation on high salt products**:** whether the country had established a tax on high salt products
6. Interventions in settings: whether there were any initiatives targeting settings including schools, hospitals, workplaces, food chains or restaurants and other institutions through education about salt and health, food procurement policy with a sodium/salt criteria, voluntary guidelines for salt/sodium levels in foods or other activities

**Evaluation**

- 1. Change in salt intake: whether the country reported changes in mean population salt intake, the assessment method used, and the baseline and follow-up salt intake
  2. Change in salt levels in foods: whether the country reported changes in salt levels of foods, the methods of measurement, and the baseline and follow-up salt levels in specific food categories
  3. Change in KAB towards salt: whether the country reported changes in consumer KAB, the methods of measurement, and the baseline and follow-up data
