# Supplementary material for: A Systematic Review of Salt Reduction Initiatives Around the World: A Midterm Evaluation of Progress Towards the 2025 Global Non-Communicable Diseases Salt Reduction Target
Source: Adv Nutr. 2021 Mar 7;12(5):1768–80. doi: 10.1093/advances/nmab008 (PMC8483946; doi:10.1093/advances/nmab008)
Supplement: nmab008_Supplemental_File [file nmab008_supplemental_file.zip › Supplementary_data_3.docx]

**Supplemental Material 2.** Country Questionnaire 2019 sent to salt reduction program leaders

**2019 POPULATION SALT REDUCTION COUNTRY QUESTIONNAIRE**

By completing this questionnaire, you consent for the information provided in this questionnaire to be used for research.

**Name of country:**

|  |
| --- |

**YOUR NAME, ORGANISATION and POSITION:**

|  |
| --- |

**YOUR CONTACT DETAILS:**

|  |
| --- |

Please tick this box if you wish to receive a copy of the results

Please answer the following questions as accurately as possible or if you prefer, please attach the relevant documents and web links that provides answers to the questions.

Information obtained prior to 2014 is in blue, new information on initiatives implemented after 2014 have been pre-filled in black.

# 1. National salt reduction initiatives

**1. Is there or has there previously been any program, policy or initiative designed to reduce population levels of salt/sodium intake in your country?** *This includes initiatives led by government, industry, non-governmental organizations (NGOs) or any other agency, and may include any of the following: regulation, product reformulation, labelling, consumer awareness/ education, dietary target development, strategy development, monitoring/surveillance, research etc.*

| **YES** *(continue question 1)* | **NO** *(go to question 2)* | **PLANNED** *(continue question 1)* |
| --- | --- | --- |

| **Name of initiative:** | | | **Time frame:** |
| --- | --- | --- | --- |
| **Is there a national target for dietary salt intake?** | YES  NO  PLANNED | If YES, what is the target? ….…... | |
| **Who is the lead agency of the national salt reduction initiative?** | Government  NGO  Food Industry  Other, please specify: ….…... | | |
| **Is the initiative part of a broader program?** | YES  NO, the initiative is a salt specific program | | |
| **Are there any non-governmental or health organizations advocating for salt reduction in the country?** | YES  NO | | |

**2. Population salt intake**

**2A. Has any work been done to measure levels of salt/sodium intake in your country?**

| **YES** *(continue question 2)* | **NO** *(go to question 2B)* | **PLANNED** *(continue question 2)* |
| --- | --- | --- |

|  | **Year** | **Method** (E.g. dietary survey, 24 hour urine collection, spot urine.) | **Salt intake** (Total average, male average & female average) |
| --- | --- | --- | --- |
| **Please provide details on all the measurements of population salt intake in the country** (Insert additional rows if needed) |  |  |  |
|  |  |  |  |
|  |  |  |  |

**2B. Has any work been done to estimate the dietary sources of salt/sodium in your country?**

| **YES** *please provide link or report name* ….…...  *OR complete the table below* | **NO** *please roughly estimate the major sources of salt in the table below* | **PLANNED** *please roughly estimate the major sources of salt in the table below* |
| --- | --- | --- |

| **Dietary sources** (E.g. packaged foods versus discretionary salt intake bread, processed meats, foods eaten out of home) | **Percent (%) contribution to total salt intake** | **Absolute contribution (salt in g/d or sodium mg/d)** | **Estimation method** (E.g. dietary recall, food frequency questionnaire, sales data, household expenditure survey etc) |
| --- | --- | --- | --- |
|  |  |  |  |
|  |  |  |  |

# 3. Salt levels in foods or meals

**Has any work been done to determine the salt/sodium levels in foods or meals?**

| **YES** *(continue question 3)* | **NO** *(go to question 4)* | **PLANNED** *(continue question 3)* |
| --- | --- | --- |

|  | **Year** | **Method** (E.g. food analysis, survey, database, industry self-report) | **Food categories collected** | **Reduction in salt/sodium content demonstrated** |
| --- | --- | --- | --- | --- |
| **Please provide details on all the measurements of salt levels in foods or food categories in the country.** (Insert additional rows if needed) |  |  |  | YES. Please specify which foods: ….…...  NO  N/A |
|  |  |  |  | YES. Please specify which foods: ….…...    NO  N/A |

# 4. Consumer knowledge, attitudes and behaviour

**Has any work been done to determine consumer knowledge, attitude and behaviour (KAB) in relation to salt/sodium?**

| **YES** *(continue question 4)* | **NO** *(go to question 5)* | **PLANNED** *(continue question 4)* |
| --- | --- | --- |

|  | **Year** | **Method** (focus group, survey) | **Improvements in consumer KAB demonstrated** |
| --- | --- | --- | --- |
| **Please provide details on all the measurements of consumer knowledge, attitudes and behaviours in relation to salt in the country** (Insert additional rows if needed) |  | Survey  Focus group  Other | YES  NO  N/A |
|  |  | Survey  Focus group  Other | YES  NO  N/A |

**5. Implementation strategies**

**5A. ENGAGEMENT WITH INDUSTRY & REFORMULATION
Does your strategy include work with industry to achieve salt/sodium reduction in foods?**

| **YES** *(continue question 5a)* | **NO** *(go to question 5b)* | **PLANNED** *(continue question 5a)* |
| --- | --- | --- |

| **Name of initiative & year implemented** |  | | |
| --- | --- | --- | --- |
| **Agency/organization taking the lead to engage industry** | Government  NGO | Industry  Other, please specify: ….…... | |
| **Is the approach voluntary or mandatory?** | Voluntary  Mandatory for all food categories (eg maximum salt content in food)  Mandatory for certain food categories such as ……………. | | |
| **Approach to work with industry** | Meetings with companies  Voluntary commitments to salt reduction from companies  Cross-sectoral agreements to salt reduction (e.g. all bread manufacturers)  Targets for salt levels in foods  Taxation for high salt products  Other, please specify ……………. | | |
| **If salt reduction targets have been used, which food categories have reformulation targets:** | Breads  Processed meats  Convenience/Ready meals  Breakfast cereals  Cheeses | | Butter and margarines  Salty snacks  Biscuits and cakes  Soups and Sauces,  Other, please specify ……………. |

**5B. CONSUMER EDUCATION/ BEHAVIOUR CHANGE
Does your strategy include activities to raise awareness/change behaviour on salt/sodium?**

| **YES** *(continue question 5b)* | **NO** *(go to question 5c)* | **PLANNED** *(continue question 5b)* |
| --- | --- | --- |

| **Name of initiative & year implemented** | **Agency / organization taking the lead** | **Approach** |
| --- | --- | --- |
|  | Government  NGO  Industry  Other, please specify: ….…... | Social marketing (e.g. campaigns)  TV advertising  Events  Other, please specify: ….…... |
|  | Government  NGO  Industry  Other, please specify: ….…... | Social marketing (e.g. campaigns)  TV advertising  Events  Other, please specify: ….…... |

**5C. NUTRITION LABELLING ON PACKAGED FOODS**

**Is it mandatory that packaged foods display the sodium or salt content as part of the nutrition information panel in your country?** *(Example of nutrition information panel shown below)*


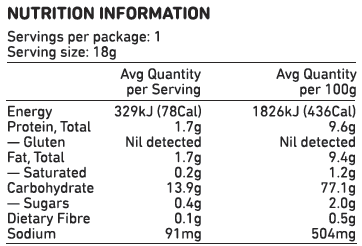


| **YES** *(continue question 5c)* | **NO** *(continue question 5c)* | **PLANNED** *(continue question 5c)* |
| --- | --- | --- |

**Has your country introduced front of pack nutrition labels to indicate the nutritional or salt/sodium content of food?** *Front of pack labels on packaged foods aim to provide consumers with simplified, easy-to-understand nutrition information to help them make healthier choices*

| **YES** *(continue question 5c)* | **NO** *(continue question 5c)* | **PLANNED** *(continue question 5c)* |
| --- | --- | --- |

| **Name of initiative and year implemented** |  | | | | |
| --- | --- | --- | --- | --- | --- |
| **Agency / organization taking the lead** | Government  Industry  NGO  Other | | | | |
| **Approach/ type of label** *(Please tick all that are applicable)* | **Rating or lights**  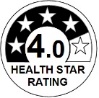**Example:** 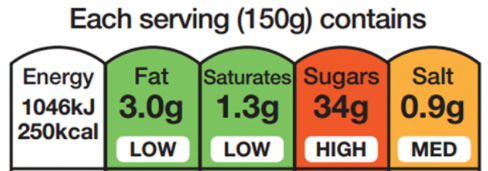 | **Nutrient-specific Warning label**  **Example:**  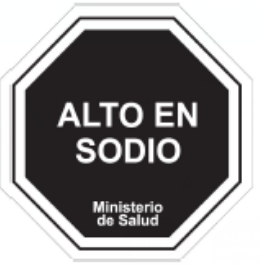 | **Percent daily intake**  **Example:**  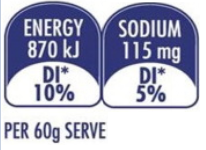 | **Health Logo/Positive summary symbol**  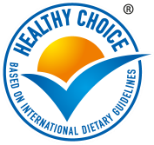**Example:** | **Other, please specify:** |
|  |  |  |  |  |  |
| **Is the corresponding approach voluntary or mandatory?** | Voluntary  Mandatory for all food categories  Mandatory for certain food categories such as ……………. | Voluntary  Mandatory for all food categories  Mandatory for certain food categories such as ……………. | Voluntary  Mandatory for all food categories  Mandatory for certain food categories such as ……………. | Voluntary  Mandatory for all food categories  Mandatory for certain food categories such as ……………. | Voluntary  Mandatory for all food categories  Mandatory for certain food categories such as ……………. |

**Does your country have any type of front-of-pack labels (voluntary or mandatory) for other nutrients (energy, saturated fat, sugar, etc)?**

| **YES** *(continue question 5c)* | **NO** *(go to question 5d)* | **PLANNED** *(continue question 5c)* |
| --- | --- | --- |

| **Nutrient** | **Approach** (voluntary of mandatory) | **Type of label** (traffic light label, health logo, warning label, percent daily intake, etc - please list all that are applicable) |
| --- | --- | --- |
|  |  |  |
|  |  |  |

**5D. WORK IN SPECIFIC SETTINGS
Does the strategy include initiatives on salt/sodium in particular settings such as schools, hospitals or workplaces?** Examples of strategies in settings can include:

A – Mandatory food procurement policy

B – Voluntary guidelines

C – Education

D – Menu labelling

E – Toolkit/implementation guide/support

F – Achievement/accreditation program

G – Others (please specify)

| **YES** *(continue question 5d)* | **NO** *(go to question 5e)* | **PLANNED** *(continue question 5d)* |
| --- | --- | --- |

| **Setting** | **Name and description of initiative & year implemented** | **Components of initiatives in particular settings** (please indicate the letter/s from choices above for each setting) |
| --- | --- | --- |
| Schools |  |  |
| Hospitals |  |  |
| The workplaces |  |  |
| Government facilities/offices |  |  |
| Food eaten away from home e.g. food outlets, restaurants, fast food chains |  |  |
| ☐ Others, please specify ….…... |  |  |

**5E. LOW-SODIUM SALT SUBSTITUTES**

**Does your country have a national position on the use of low-sodium salt substitutes (e.g. potassium enriched salt)?**

| **YES** *(continue question 5e)* | **NO** *(go to question 6)* | **PLANNED** *(continue question 5e)* |
| --- | --- | --- |

| **Name of initiative & year implemented** |  |
| --- | --- |
| **Agency / organization taking the lead** | Government  Industry  NGO  Other |
| **Approach** | Commercially available for consumers to purchase from shops  Recommended through health practitioners for those with pre-hypertension or hypertension  Recommended to food manufacturers as a method to lower sodium content in food products  Other, please specify ….…... |

# 6. Evaluation of salt/sodium reduction strategy

**Has your country done any work to evaluate the effectiveness of the salt/sodium reduction strategy or any component of the strategy, in addition to monitoring salt intake, salt levels and consumer KAB? (This can also include process evaluation or economic evaluation)**

| **YES** *(continue question 6)* | **NO** *(go to question 7)* | **PLANNED** *(continue question 6)* |
| --- | --- | --- |

**For each evaluation, please specify:**

(please copy and paste the table below as needed, one table per evaluation)

| **Year evaluated** |  |
| --- | --- |
| **Evaluation approach** |  |
| **Results  (Has it had an impact?)** |  |
| **Has a cost-effective analysis been undertaken?** | YES  NO  PLANNED |
| **What was the result of the cost-effectiveness assessment?** |  |

# 7. National initiatives to increase iodine and potassium intake

**7A. Is there or has there previously been any program, policy or initiative designed to eliminate iodine deficiency in your country?**

| **YES, in coordination with the salt reduction strategy** *(continue question 7A)* | **YES, but NOT in coordination with the salt reduction strategy** *(continue question 7A)* | **NO** *(go to question 7B)* | **PLANNED** *(continue question 7A)* |
| --- | --- | --- | --- |

| **Name of initiative & year implemented** |  |
| --- | --- |
| **Agency / organization taking the lead** | Government  Industry  NGO  Other  Contact name and email: ….…... |
| **Population approaches taken** | Universal salt iodization (mandatory iodization of all salt for human consumption)  Mandatory iodized salt use in certain foods or beverages  Voluntary salt iodization  Other, please specify: ….…... |
| **Approaches taken to integrate salt reduction and iodine deficiency elimination programs** | Integrated communication/education for example: “eat less salt and only iodized”  Monitor salt and iodine intake concurrently (through urine or dietary survey)  Adapt iodine levels in salt as salt intake decreases  None yet  Other, please specify: ….…... |

**7B. Is there or has there previously been any program, policy or initiative designed to increase potassium intake in your country?**

| **YES, in coordination with the salt reduction strategy** *(continue question 7B)* | **YES, but NOT in coordination with the salt reduction strategy** *(continue question 7B)* | **NO** *(end of survey)* | **PLANNED** *(continue question 7B)* |
| --- | --- | --- | --- |

| **Name of initiative & year implemented** |  |
| --- | --- |
| **Agency / organization taking the lead** | Government  Industry  NGO  Other  Contact name and email: ….…... |
| **Population approaches taken** | Potassium-enriched, low-sodium salt substitute  Healthy eating plan (for example recommended intake of foods that are high in potassium)  National dietary guidelines (for example recommended daily intake of potassium)  Fortification of potassium in foods  Other, please specify: ….…... |

Please attach any documents and website links relevant to the questions.

**THANK YOU FOR COMPLETING THIS QUESTIONNAIRE.** We value your time. Please send the completed questionnaire and relevant documents to Joseph Santos on [jsantos@georgeinstitute.org.au](mailto:jsantos@georgeinstitute.org.au).
