# Supplementary material for: A Systematic Review of Salt Reduction Initiatives Around the World: A Midterm Evaluation of Progress Towards the 2025 Global Non-Communicable Diseases Salt Reduction Target
Source: Adv Nutr. 2021 Mar 7;12(5):1768–80. doi: 10.1093/advances/nmab008 (PMC8483946; doi:10.1093/advances/nmab008)
Supplement: nmab008_Supplemental_File [file nmab008_supplemental_file.zip › Supplementary_data_4.docx]

**Supplemental Table 2.** Implementation strategies and reported data on change of the 96 National Salt Reduction Initiatives^1^

| **COUNTRY (Supplemental reference)** | **IMPLEMENTATION STRATEGIES** | | | | **REPORTED DATA ON CHANGE**^2^ | | |
| --- | --- | --- | --- | --- | --- | --- | --- |
|  | **Food reformulation** | **Interventions in settings**^3^ | **Front-of-pack labelling** | **Consumer education** | **Salt intake** | **Salt levels in foods** | **KAB towards salt** |
| **Argentina** (1-8) | Targets (vol/man) | PP/VG (sch/wk)  ML/O (rest) | Planned (man) | Gov | 2011 to 2015  24-hour urine  11.2g/d to 9.2g/d | 2011/12; 2014/15; 2017/18  Food label survey and food analysis | - |
| **Australia** (9-13) | Targets (vol) | VG (sch/wk/hosp) | HSR (vol) | - | 2011 to 2014  24-hour urine  7.9g/d to 7.8g/d | 2009/12 to 2015  Database | 2011 to 2014  Survey |
| **Austria** (14-16) | Targets (vol) | VG (sch) | HM (vol) | - | 2008 to 2012  24-hour food recall  8.3g/d to 8.2g/d | 2011 to 2013  Industry self-report | - |
| **Bahrain** (5, 10, 17, 18) | Targets (man) (bread) | E/PP (sch) | Planned (bread) | - | - | - | - |
| **Bangladesh** (5), Q2019 | Planned IM | Planned E (sch/wk) | - | Gov/NGO | - | - | - |
| **Barbados** (7, 8, 19),  Q2019 | IM/VA | E/AP (sch)  E/O (rest)  VG (sch/wk) | Planned WL (man) | Gov | - | - | - |
| **Belarus** (5) | Targets (man) | - | - | - | - | - | - |
| **Belgium** (10, 14, 15, 20, 21), Q2019 | Targets (vol/man) | VG (sch/rest) | Logo (vol) | - | - | 2009 to 2013  Method unspecified | - |
| **Bermuda** (10) | - | PP (sch/gov) | - | - | - | - | - |
| **Bhutan** (5) | Planned IM | Planned E/PP (sch/wk) | - | Gov | - | - | - |
| **Bolivia, Plurinational State of** (5, 7, 8, 22) | - | O (rest)^4^ | TL/HM (man)^4^ | - | - | - | - |
| **Brazil** (7, 8, 23, 24),  Q2019 | Targets (vol) | E/PP/VG (sch/wk)  VG (rest/gov) | Planned WL (man) | Gov | - | 2011 to 2017  Food label survey | - |
| **Bulgaria** (14, 15) | Targets (vol/man) | PP (sch) | % DI (vol) | Gov | - | - | - |
| **Canada** (7, 8, 16, 25-28) | Targets (vol) | VG (sch/wk/hosp/o)  ML (rest)  AP (sch/wk/o) | Logo (vol)  Planned WL (man) |  | 2004 to 2015  24-hour food recall  8.5g/d to 6.9g/d | 2012 to 2017  Food label survey | - |
| **Chile** (7, 8, 29-32)  Q2019 | Targets (vol) | PP (sch) | WL (man) | Gov | - | 2010 to 2012  Food analysis | - |
| **China, People’s Republic of** (5, 33-35) | - | E (sch/rest/o) | % DI (vol) | Gov | 2000 to 2009/12  Total diet study  11.8g/d to 9.1g/d | - | 2012  Survey |
| **Colombia** (7, 8, 36, 37) | Targets (vol) | - | Planned WL (man) | Gov | - | - | - |
| **Cook Islands** (5) | Targets (vol)  Planned tax | PP (sch/wk)  Planned O (rest) | - | Planned Gov | - | - | - |
| **Costa Rica** (7, 8, 10, 30, 38, 39), Q2019 | Targets (vol) | PP (sch)  ML (rest)  Planned O (rest) | % DI (vol) | Gov | - | 2015 to 2018  Food label survey | - |
| **Croatia** (14, 21, 40) | Targets (vol) (bread) | PP (sch) | Logo (vol) | - | - | - | 2008 to 2017  Survey |
| **Cyprus** (14) | - | E/VG (sch) | Yes (vol) | - | - | - | - |
| **Czech Republic** (15, 21) | Targets (vol) | - | Logo (vol) | - | - | - | - |
| **Denmark** (14, 20, 21, 41), Q2019 | Targets (vol) | VG (sch/wk) | Logo (vol) | Gov | 2006 to 2010  Spot urine  M: 10.7g/d to 9.9g/d  F: 7.5g/d to 7.0g/d | - | - |
| **Ecuador** (7, 8, 10, 42, 43) | Targets (vol) | PP (sch) | TL (man) | - | - | - | - |
| **Egypt** (5) | IM (bread)  Planned targets (man) | E (rest) | - | - | - | - | - |
| **El Salvador** (7, 8, 44, 45) | Planned targets | PP (sch)  Planned E/ML (rest)  Planned E (wk/hosp/gov/o) | Planned WL | Planned Gov | - | - | - |
| **Estonia** (10, 14) | - | PP (sch/hosp) | % DI (vol) | - | - | - | - |
| **Fiji** (10, 46-48)  Q2019 | Targets (vol)  Tax (MSG) | E (sch/wk/hosp/gov)  VG (sch/hosp) | Planned HSR (vol) | Gov | 2012/13 to 2015/16  24-hour urine  11.7g/d to 10.3g/d | 2012/13 to 2015/16  Food label survey | 2012/13 to 2015/16  Survey |
| **Finland** (14, 16, 20, 21, 32, 49, 50) | Targets (vol/man) | PP (sch)  VG (wk/hosp/gov/o) | Logo (vol)  WL (man) | - | 1979 to 1987  24-hour urine  M: 13.1 to 12.0g/d  F: 10.4 to 9.5g/d  1979 to 2002  M: 12.9 to 9.5g/d  F: 10.4 to 7.4g/d  1982 to 2002  M: 11.6 to 9.8g/d  F: 9.1 to 7.4g/d | 1990s to 2009  Food analysis | - |
| **France** (14, 16, 21, 51) | Targets (vol) | PP (sch) | NS (vol) | - | 1998/99 to 2015/16  7-day food record  8.0g/d to 7.5g/d | 2008/10 to 2011/13  Database and HCPD | - |
| **French Polynesia** (5), Q2019 | VA (bread)  Planned tax | E (sch)  VG/IG (wk) | - | Gov | - | - | - |
| **Georgia** (52, 53), Q2019 | Planned IM/targets | - | % DI (vol) | Gov/NGO | - | - | - |
| **Germany** (10, 20, 54), Q2019 | Targets (vol)  VA | VG (sch/wk/o)  Planned VG (rest) | - | Gov | - | - | - |
| **Greece** (14, 55), Q2019 | Targets (vol/man) | E/PP (sch)  O (rest) | % DI (vol) | Gov/NGO | - | - | - |
| **Guam** (10, 56, 57) | - | PP (sch/gov) | - | Planned Gov | - | - | - |
| **Hong Kong** (5, 10, 58) | IM/Planned targets (vol) | E/VG (sch)  E/ML/O (rest) | HM (vol) | Gov | - | - | - |
| **Hungary** (14, 20, 59) | Targets (vol/man)  Tax | PP (sch) | - | NGO | - | - | 2011 to 2014  Survey |
| **Iceland** (21, 41, 60, 61) | - | PP (sch) | Logo (vol) | - | 2002 to 2010/11  24-hour food recall  8.4g/d to 7.9g/d | - | - |
| **Indonesia** (5, 62, 63) | Planned IM/tax | - | HM (man)  % DI (vol) | - | - | - | - |
| **Iran, Islamic Republic of** (10, 64-67) | Targets (man) | PP (sch) | TL (man) | - | - | - | - |
| **Ireland** (14, 41, 68, 69), Q2019 | Targets (vol) | VG (sch) | % DI (vol) | NGO | 2001 to 2011  4-day food record  8.1g/d to 7.0g/d | 2003 to 2018  Food analysis | 2011 to 2017  Consumer survey |
| **Israel** (10, 21) | Targets (vol) | PP (sch)  Planned VG (wk/gov) | WL (man)  Logo (vol) | - | - | - | - |
| **Italy** (14, 55, 70), Q2019 | Targets (vol) | E/VG (sch) | - | Gov/NGO | 2009/10 to 2012  24-hour urine  M: 10.6g/d to 9.3g/d  F: 8.3g/d to 7.2g/d | 2015  Method unspecified | 2009/10 to 2012  Survey |
| **Japan** (71, 72) | VA (noodles) (FI) | - | - | - | 2011 to 2017  Dietary records  9.9g/d to 9.4g/d | - | - |
| **Jordan** (10, 64, 73) | IM/VA (bread)  Planned targets (man) (bread) | PP (sch) | - | - | - | - | - |
| **Korea, Republic of (South)** (5, 10, 74, 75) | Targets (vol)  VA | PP/O (sch)  ML (rest) | % DI (vol)  TL (vol) | Gov | 2005 to 2015  Dietary survey  13.2g/d to 9.7g/d | 2015  Method unspecified | Year unknown  Survey |
| **Kuwait** (5, 10, 76) | Targets (vol) | PP (sch)  Planned PP (wk/hosp/gov) | - | Gov | - | 2013  Method unspecified | - |
| **Latvia** (10, 15) | - | PP (sch/hosp/o) | % DI (vol) | - | - | - | - |
| **Lebanon** (77) | IM/Planned VA | Planned E/O (rest) | Planned | Gov/NGO | - | - | - |
| **Lithuania** (10, 21) | Targets (vol) | PP (sch) | Logo (vol) | - | 2007 to 2013/14  24-hour food recall  8.8g/d to 7.1g/d | - | - |
| **Macao** (5) | - | E/O (hosp) | - | Gov | - | - | - |
| **Malaysia** (10, 32), Q2019 | VA | PP (sch/hosp/gov/rest)  VG (wk) | Logo (vol) | GOV/NGO/FI/media | - | 2011 to 2012  Industry self-report | - |
| **Malta** (10, 14),  Q2019 | IM/VA (bread) | PP (sch/hosp) | - | Gov | - | - | - |
| **Mexico** (7, 8, 10, 30, 78, 79) | Targets (vol)  Tax | PP (sch) | % DI (man)  WL (man)  Logo (vol) | - | - | - | - |
| **Moldova, Republic of** (80, 81) | Planned targets (man) (bread) | PP/O (sch) | - | Gov | - | - | - |
| **Mongolia** (82),  Q2019 | Targets (vol) | E/VG/IG/O (sch/wk/hosp) | TL (vol)  % DI (vol) | Gov |  | 2019  Food analysis |  |
| **Montenegro** (5) | Planned targets (vol) | E (sch/wk/hosp/gov)  Planned VG (sch/wk/hosp/gov/o) | - | Planned Gov | - | - | - |
| **Morocco** (5, 64, 83, 84), Q2019 | VA (bread) | PP (sch)  E (rest)  Planned E (sch/wk/hosp) | Planned logo (man) | Gov/NGO/O | - | 2011 to 2016  Food analysis | 2012 to 2016  Survey |
| **Nauru** (5) | - | - | - | Gov | - | - | - |
| **Netherlands** (14, 20, 85), Q2019 | Targets (vol/man) | VG/O (sch/wk/hosp/gov/rest) | % DI (vol) | NGO/FI/O | 2006 to 2015  24-hour urine  M: 9.9g/d to 9.7g/d  F: 7.9g/d to 7.4g/d | 2011 to 2016  Food label survey and food analysis | 2008 to 2011  Consumer survey |
| **New Zealand** (86-88), Q2019 | Targets (vol) (NGO) | - | HSR (vol)  % DI (vol) | - | 2009 to 2016  Total diet study  M: 7.3g/d to 7.2g/d  F: 5.1g/d to 5.2g/d | 2014 to 2016  Food label survey and HCPD | - |
| **North Macedonia** (10), Q2019 | Planned targets (vol) (bread) | PP/IG (sch) | - | Planned Gov | - | - | - |
| **Norway** (14, 21, 89, 90), Q2019 | Targets (vol) | VG (sch)  E/VG/IG (wk/rest) | Logo (vol) | Gov | - | 2014/15 to 2018  Food analysis | 2015 to 2018  Survey |
| **Oman** (73, 91, 92) | VA/Targets (vol) (bread)  Planned targets (man) (bread) | - | - | Planned Gov | - | 2015  Industry self-report | - |
| **Palau** (5) | Planned tax | E/PP (sch) | - | Planned Gov | - | - | - |
| **Palestine** (93) | Targets (man) (bread) | - | - | - | - | - | - |
| **Panama** (7, 8, 44, 94) | Planned targets | PP (sch)  Planned E/ML (rest)  Planned E (wk/hosp/gov/o) | Planned WL | Planned Gov | - | - | - |
| **Paraguay** (7, 8, 42), Q2019 | Targets (man) (bread) | E (wk)  O (rest) | - | Gov | - | - | - |
| **Peru** (7, 8, 95, 96) | - | E/PP (sch) | WL (man) | Gov | - | - | - |
| **Poland** (10, 21) | Targets (vol) | PP (sch) | Logo (vol)  GDA (vol) | - | - | - | - |
| **Portugal** (55, 89, 97) | Targets (vol/man) | VG (sch)  PP (gov) | TL (vol) | Planned Gov | Year and method unspecified  1.7g/d reduction | Year and method unspecified | 2007 to 2009  Survey |
| **Qatar** (17, 64, 98) | Targets (vol/man) | - | - | Gov | - | 2014  Food analysis | - |
| **Romania** (10) | - | PP (sch) | - | Gov | - | - | - |
| **Russia** (99) | - | - | TL (vol) | Planned Gov | - | - | - |
| **Saint Vincent and the Grenadines** (5, 100) | Planned targets (vol)/Tax | Planned E/PP/O (sch) | - | Gov | - | - | - |
| **Samoa** (101, 102) | Planned targets (man) | E (sch/wk/hosp/gov/o)  VG (sch) | - | Gov | 2013 to 2015  24-hour urine  7.3g/d to 7.5g/d | 2013 to 2015  Food label survey | 2013 to 2015  Survey |
| **Saudi Arabia** (5, 17, 64, 84) | Targets (vol) (bread) | Planned E (sch/rest/o) | TL (vol) | - | - | - | - |
| **Singapore** (10, 103), Q2019 | Targets (vol) | E/VG/O (sch)  VG (wk/o) | Logo (vol) | Gov | 1998 to 2018  Dietary survey  9.0g/d to 9.0g/d | - | 1998 to 2010  Survey |
| **Slovakia** (14, 20) | Targets (man) | PP (sch) | - | - | - | - | - |
| **Slovenia** (10, 21, 89, 104), Q2019 | Targets (vol) | E/PP/IG (sch)  VG (wk/hosp) | Logo (vol)  Planned TL (vol) | Gov | 2007 to 2012  24-hour urine  M: 14.3 to 12.9g/d  F: 11.0 to 10.7g/d | 2011 to 2015  Food label survey and sales data | 2010 to 2011  Telephone interview |
| **South Africa** (105-107) | Targets (man) | - | Logo (vol) | NGO | - | 2013 to 2018  Food label survey | 2014 to 215  Survey |
| **Spain** (14, 108), Q2019 | Targets (vol/man) | PP/IG (sch)  O (rest) | Planned NS (vol) | Gov | - | 2009 to 2012  Food analysis | - |
| **Sri Lanka** (5),  Q2019 | Planned IM | VG (sch/wk/hosp/gov) | TL (man) | Gov | - | - | - |
| **Sweden** (10, 21), Q2019 | Targets (vol) | PP (sch) | Logo (vol) | - |  |  |  |
| **Switzerland** (14, 16, 109) | Targets (vol) | VG (sch/wk/hosp/gov) | Planned NS (vol) | Gov | 1984 to 2011  24-hour urine  8.4g/d to 9.2g/d | 2011 to 2015  Food analysis | - |
| **Thailand** (5, 10, 110, 111) | Planned targets (man)/Planned tax | E (sch/hosp)  VG (hosp/o)  Planned E (gov) | HM (man)  GDA (man)  Logo (vol) | Planned Gov | - | - | - |
| **Tonga** (112) | Tax | - | - | - | - | - | 2014/15 to 2017/18  Survey |
| **Tunisia** (64, 84) | Targets (vol) (bread) | - | Planned logo (man) | - | - | - | - |
| **Turkey** (113) | Targets (vol) | O (sch) | Planned logo | - | 2008 to 2012  24-hour urine  18.0g/d to 15.0g/d |  |  |
| **Turkmenistan** (114) | Targets (man) (bread) | - | - | - | - | - | - |
| **United Arab Emirates** (10, 17, 73, 84, 115, 116) | IM/VA  Planned targets (bread) | PP (sch)  ML (rest) | TL (vol)  Logo (vol) | Gov | - | - | - |
| **United Kingdom** (10, 21, 117-121), Q2019 | Targets (vol) | PP/O (sch/hosp/gov/o)  IG (wk/rest) | TL (vol)  % DI (vol) | Gov | 2005/06 to 2018/19  24-hour urine  8.1g/d to 7.5g/d | 2006 to 2011  HCPD | 2004 to 2009  Survey |
| **United States of America** (7, 8, 10, 122-126), Q2019 | Targets (vol) | E/VG/IG (sch/wk/hosp/gov/rest)  AP (hosp/rest)  ML (rest, NYC) | - | Gov/NGO | 2011/12 to 2015/16  24-hour food recall  8.7g/d to 8/5g/d | 2009 to 2014  Food label survey and sales data | 2012 to 2015  Survey |
| **Uruguay** (7, 8, 10, 15, 37) | Targets (vol) | PP (sch)  O (sch/rest) | WL (man) | Gov | - | - | - |
| **Uzbekistan** (127), Q2019 | Targets (man) | Planned E/O (sch) | Planned WL (man)  Planned logo (vol) | Gov | - | - | - |
| **Viet Nam** (128, 129), Q2019 | Targets (vol) | VG (sch/wk)  E/O (sch) | Planned logo (vol) | Gov | - | - | - |

“ – “ indicates that we are unaware of activity.

^1^ *Abbreviation list for each column:*
(1) Country: Q2019, Questionnaire 2019 as additional source.

(2) Food reformulation: FI, food industry; IM, industry meetings; man, mandatory; MSG, monosodium glutamate; NGO, non-governmental organization; VA, voluntary agreements; vol, voluntary.

(3) Interventions in settings: AP, achievement or accreditation program; E, education; gov, government offices; hosp, hospitals; IG, implementation guide; ML, menu labelling; o, other settings; O, others; PP, procurement policy; rest, restaurants; sch, schools; VG, voluntary guidelines; wk, workplaces.

(4) Front-of-pack labelling: % DI, percent daily intake; GDA, guideline daily amount; HM, health messages; HSR, health star rating; man, mandatory; NS, nutri-score; TL, traffic light label; vol, voluntary; WL, warning label.

(5) Consumer education: FI, food industry; gov, government; NGO, non-governmental organization.

(6) Salt intake: F, females; g/d, grams per day; M, males.

(7) Salt levels in foods: HCPD, household consumer panel data.

^2^ Reported data on change: includes data on change in salt intake, salt levels in foods, and knowledge, attitudes and behaviours towards salt, measured using comparable assessment methods over time

^3^ Interventions in settings: includes interventions at subnational level (i.e. local, provincial, or state-level)

^4^ In progress

**SUPPLEMENTAL REFERENCES**

1. El Senado y Cámara de Diputados de la Nación Argentina reunidos en Congreso. Alimentos ley 26.905 consumo de sodio. Valores máximos. 2013.

2. Ministerio de Justicia y Derechos Humanos. Reglamentación de la ley 26.905 sobre promoción de la reducción del consumo de sodio en la población. 2017.

3. International Food Policy Research Institute. Global nutrition report 2016: From promise to impact: Ending malnutrition by 2030. Washington, DC 20006-1002 USA: International Food Policy Research Institute, 2016.

4. Calliope SR, Sammán N. 12th ifdc 2017 special issue –sodium content in foods consumed by argentines: Monitoring compliance with agreements, in labels and samples. Journal of Food Composition and Analysis. 2019;83:103289. doi: <https://doi.org/10.1016/j.jfca.2019.103289>.

5. World health organization noncommunicable disease document repository [Internet]. 2020. Available from: <https://extranet.who.int/ncdccs/documents/default>.

6. Fundacion InterAmericana del Corazon-Argentina. Monitoreo del contenido de sodio en productos procesados de argentina 2018-2018. Argentina: Fundacion InterAmericana del Corazon-Argentina, 2019.

7. Pan American Health Organization. Mapping dietary sodium reduction policies and initiatives in the region of the americas (unpublished data). 2020.

8. Flexner N, L'Abbé M, Legowski B, Toledo RG. Mapping dietary sodium reduction policies and initiatives in the region of the americas. Current Developments in Nutrition. 2020;4(Supplement_2):1714-. doi: 10.1093/cdn/nzaa064_004 %J Current Developments in Nutrition.

9. Jones A, Magnusson R, Swinburn B, Webster J, Wood A, Sacks G, Neal B. Designing a healthy food partnership: Lessons from the australian food and health dialogue. BMC Public Health. 2016;16:651. Epub 2016/07/29. doi: 10.1186/s12889-016-3302-8.

10. World Cancer Research Fund International. Nourishing framework: Offer healthy food and set standards in public institutions and other specific settings. London, UK: World Cancer Research Fund International, 2019.

11. Magnusson R, Reeve B. Food reformulation, responsive regulation, and "regulatory scaffolding": Strengthening performance of salt reduction programs in australia and the united kingdom. Nutrients. 2015;7(7):5281-308. Epub 2015/07/03. doi: 10.3390/nu7075221.

12. Nowson C, Lim K, Grimes C, O'Halloran S, Land MA, Webster J, Shaw J, Chalmers J, Smith W, Flood V, et al. Dietary salt intake and discretionary salt use in two general population samples in australia: 2011 and 2014. Nutrients. 2015;7(12):10501-12. Epub 2015/12/24. doi: 10.3390/nu7125545.

13. National Heart Foundation of Australia. Report on the evaluation of the nine food categories for which reformulation targets were set under the food and health dialogue. ACT, Australia: National Heart Foundation of Australia, 2016.

14. European Commission Joint Research Centre, Institute for Health and Consumer Protection. Mapping of national school food policies across the eu28 plus norway and switzerland. Luxembourg: Publications Office of the European Union, 2014.

15. Center for Science in the Public Interest. International action on sodium. Washington, DC 20005, USA: Center for Science in the Public Interest, 2016.

16. Barberio AM, Sumar N, Trieu K, Lorenzetti DL, Tarasuk V, Webster J, Campbell NRC, McLaren L. Population-level interventions in government jurisdictions for dietary sodium reduction: A cochrane review. Int J Epidemiol. 2017;46(5):1551-405. Epub 2017/02/17. doi: 10.1093/ije/dyw361.

17. Alhamad N, Almalt E, Alamir N, Subhakaran M. An overview of salt intake reduction efforts in the gulf cooperation council countries. Cardiovasc Diagn Ther. 2015;5(3):172-7. Epub 2015/06/20. doi: 10.3978/j.issn.2223-3652.2015.04.06.

18. Kingdom of Bahrain Ministry of Health. Moh organize a workshop for school canteen operators in collaboration with school health section at the ministry of education 2019. Available from: <https://www.moh.gov.bh/News/Details/3453?lang=en>.

19. Healthy Caribbean Coalition. A civil society report on national ncd commissions in the caribbean: Towards a more effective multisectoral response to ncds part i. Healthy Caribbean Coalition, 2015.

20. Lloyd-Williams F, Bromley H, Orton L, Hawkes C, Taylor-Robinson D, O’Flaherty M, McGill R, Anwar E, Hyseni L, Moonan M, et al. Smorgasbord or symphony? Assessing public health nutrition policies across 30 european countries using a novel framework. BMC Public Health. 2014;14(1):1195. doi: 10.1186/1471-2458-14-1195.

21. Kelly B, Jewell J. What is the evidence on the policy specifications, development processes and effectiveness of existing front-of-pack food labelling policies in the who european region? Copenhagen: WHO Regional Office for Europe, 2018.

22. Robles-Valcarcel P. An update on the status of front-of-package labelling regulations in latin america. Public Health Nutrition. 2017;20(5):948-9. Epub 2016/12/20. doi: 10.1017/S1368980016003359.

23. Nilson EA. The strides to reduce salt intake in brazil: Have we done enough? Cardiovasc Diagn Ther. 2015;5(3):243-7. Epub 2015/06/20. doi: 10.3978/j.issn.2223-3652.2015.04.03.

24. Nilson EAF, Spaniol AM, Goncalves VSS, Moura I, Silva SA, L'Abbe M, Jaime PC. Sodium reduction in processed foods in brazil: Analysis of food categories and voluntary targets from 2011 to 2017. Nutrients. 2017;9(7). Epub 2017/07/15. doi: 10.3390/nu9070742.

25. Health Canada. Toward front-of-package nutrition labels for canadians: Consultation document. Canada: Health Canada, 2016.

26. The Conference of Provincial-Territorial Ministers of Health. Reducing the sodium intake of canadians: A provincial and territorial report on progress and recommendations for future action. Canada: The Conference of Provincial-Territorial Ministers of Health, 2012.

27. Health Canada. Sodium reduction in processed foods in canada: An evaluation of progress toward voluntary targets from 2012 to 2016. Ottawa, ON K1A OK9, Canada: Health Canada, 2018.

28. Health Canada. Sodium intake of canadians in 2017. Ottawa, ON K1A 0K9, Canada: Health Canada, 2018.

29. Kanter R, Reyes M, Swinburn B, Vandevijvere S, Corvalán C. The food supply prior to the implementation of the chilean law of food labeling and advertising. Nutrients. 2018;11(1). Epub 2019/01/02. doi: 10.3390/nu11010052.

30. Instituto Nacional de Salud Pública de México. Review of current labelling regulations and practices for food and beverage targeting children and adolescents in latin america countries (mexico, chile, costa rica and argentina) and recommendations for facilitating consumer information. United Nations Children’s Fund, UNICEF, 2016.

31. Taillie LS, Reyes M, Colchero MA, Popkin B, Corvalán C. An evaluation of chile's law of food labeling and advertising on sugar-sweetened beverage purchases from 2015 to 2017: A before-and-after study. PLoS Med. 2020;17(2):e1003015. Epub 2020/02/12. doi: 10.1371/journal.pmed.1003015.

32. Webster J, Trieu K, Dunford E, Hawkes C. Target salt 2025: A global overview of national programs to encourage the food industry to reduce salt in foods. Nutrients. 2014;6(8):3274-87. Epub 2014/09/10. doi: 10.3390/nu6083274.

33. Shao S, Hua Y, Yang Y, Liu X, Fan J, Zhang A, Xiang J, Li M, Yan LL. Salt reduction in china: A state-of-the-art review. Risk Manag Healthc Policy. 2017;10:17-28. Epub 2017/03/07. doi: 10.2147/rmhp.S75918.

34. Hipgrave DB, Chang S, Li X, Wu Y. Salt and sodium intake in china. JAMA. 2016;315(7):703-5. doi: 10.1001/jama.2015.15816 %J JAMA.

35. Zhang J, Astell-Burt T, Seo DC, Feng X, Kong L, Zhao W, Li N, Li Y, Yu S, Feng G, et al. Multilevel evaluation of 'china healthy lifestyles for all', a nationwide initiative to promote lower intakes of salt and edible oil. Prev Med. 2014;67:210-5. Epub 2014/08/05. doi: 10.1016/j.ypmed.2014.07.019.

36. Ministry of Health and Social Protection. Progress to achieve reduction in salt intake: Ministry of Health and Social Protection; 2015 [cited 2019 December]. Available from: <https://www.minsalud.gov.co/English/Paginas/Progress-to-achieve-reduction-in-salt-intake.aspx>.

37. Mora-Plazas M, Gómez LF, Miles DR, Parra DC, Taillie LS. Nutrition quality of packaged foods in bogotá, colombia: A comparison of two nutrient profile models. Nutrients. 2019;11(5). Epub 2019/05/08. doi: 10.3390/nu11051011.

38. World Action on Salt and Health. Costa rica 2016. Available from: <http://www.worldactiononsalt.com/worldaction/southamerica/costa-rica/>.

39. Vega-Solano J, Blanco-Metzler A, Benavides-Aguilar KF, Arcand J. An evaluation of the sodium content and compliance with the national sodium reduction targets among packaged foods sold in costa rica in 2015 and 2018. Nutrients. 2019;11(9). Epub 2019/09/22. doi: 10.3390/nu11092226.

40. Domislovic V, Dapic K, Milicic B, Matasin M, Bukal N, Capak K, Drenjancevic I, Gulin M, Cavrak VH, Jelakovic A, et al. Positive trends in awareness of harmful effects of high salt intake - 10 years croatian action on salt and health (crash). Data from 2008 and 2017 world hypertension days. 2018;36:e130. doi: 10.1097/01.hjh.0000539338.99882.af.

41. Trieu K, Neal B, Hawkes C, Dunford E, Campbell N, Rodriguez-Fernandez R, Legetic B, McLaren L, Barberio A, Webster J. Salt reduction initiatives around the world – a systematic review of progress towards the global target. PLOS ONE. 2015;10(7):e0130247. doi: 10.1371/journal.pone.0130247.

42. Campbell N, Legowski B, Legetic B, Ferrante D, Nilson E, Campbell C, L'Abbé M. Targets and timelines for reducing salt in processed food in the americas. J Clin Hypertens (Greenwich). 2014;16(9):619-23. Epub 2014/08/01. doi: 10.1111/jch.12379.

43. Freire WB, Waters WF, Rivas-Mariño G, Nguyen T, Rivas P. A qualitative study of consumer perceptions and use of traffic light food labelling in ecuador. Public Health Nutr. 2017;20(5):805-13. Epub 2016/09/14. doi: 10.1017/s1368980016002457.

44. Instituto de Nutricion de Centro America y Panama (INCAP), Organizacion Panamericana de la Salud/Organizacion Mundial de la Salud (OPS/OMS), Consejo de Ministros de Salud de Centro America y Republica Dominicana (COMISCA). Estrategia regional para la reduccion del consumo de sal y sodio en centroamerica y republica dominicana. 2019.

45. Centro de Documentacion Judicial. Acuerdo nº 733 - normativa de tiendas y cafetines escolares saludables. 2017.

46. Pacific Research Centre for the Prevention of Obesity and NCDs (C-POND). Impact of food-related taxes in fiji. Suva, Fiji: Pacific Research Centre for the Prevention of Obesity and NCDs (C-POND).

47. Webster J, Pillay A, Suku A, Gohil P, Santos JA, Schultz J, Wate J, Trieu K, Hope S, Snowdon W, et al. Process evaluation and costing of a multifaceted population-wide intervention to reduce salt consumption in fiji. Nutrients. 2018;10(2). Epub 2018/02/02. doi: 10.3390/nu10020155.

48. Pillay A, Trieu K, Santos JA, Sukhu A, Schultz J, Wate J, Bell C, Moodie M, Snowdon W, Ma G, et al. Assessment of a salt reduction intervention on adult population salt intake in fiji. Nutrients. 2017;9(12). Epub 2017/12/13. doi: 10.3390/nu9121350.

49. Food for health: Finnish nutrition policy in action. Kuusipalo H, Maattanen-Bourke O, editors. Finland: Ministry of Social Affairs and Health, Ministry of Agriculture and Forestry.

50. Laatikainen T, Pietinen P, Valsta L, Sundvall J, Reinivuo H, Tuomilehto J. Sodium in the finnish diet: 20-year trends in urinary sodium excretion among the adult population. European Journal of Clinical Nutrition. 2006;60(8):965-70. doi: 10.1038/sj.ejcn.1602406.

51. Spiteri M, Soler LG. Food reformulation and nutritional quality of food consumption: An analysis based on households panel data in france. Eur J Clin Nutr. 2018;72(2):228-35. Epub 2017/12/23. doi: 10.1038/s41430-017-0044-3.

52. Ministry of Health, Labour and Social Affairs, National Center for Disease Control and Public Health. National strategy and action plan for non-communicable diseases prevention and control 2017-2020. Tbilisi, Georgia: Ministry of Health,, Labour and Social Affairs, National Center for Disease Control and Public Health.

53. Webb E. Nutrition labels are changing, gradually. Georgia Health News. 2018.

54. Gerlach S, Joost H. German reduction strategy on salt, sugar and saturated fat (2016). Ernaehrungs Umschau. 2016;63(4):90-3. doi: 10.4455/eu.2016.020.

55. World Health Organization Regional Office for Europe. Meeting of the who action network on salt reduction in the population in the european region (esan): Meeting report 20-21 april 2016, lisbon, portugal. World Health Organization Regional Office for Europe, 2016.

56. Reduce: Take the salt reduction challenge for healthier lives. Pacific Daily News. 2015.

57. Wilson M, Linke L, Bellhouse-King M, Singh M. Nutrition and physical education policy and practice in pacific region secondary schools. (issues & answers report, rel 2012–no. 117). Washington, DC: U.S.: Department of Education, Institute of Education Sciences, National Center for Education Evaluation and Regional Assistance, Regional Educational Laboratory Pacific, 2011.

58. Department of Health, Food and Health Bureau. Towards 2025 strategy and action plan to prevent and control non-communicable diseases in hong kong. Target 4 reduce salt intake. Hong Kong: Department of Health, 2018.

59. World Health Organization Regional Office for Europe. Assessment of the impact of a public health product tax. Copenhagen, Denmark: World Health Organization Regional Office for Europe, 2015.

60. World Health Organization Regional Office for Europe. Mapping salt reduction initiatives in the who european region. Copenhagen, Denmark: World Health Organization Regional Office for Europe, 2013.

61. Gisladottir E. School meal guidelines in iceland: Nordic school meal meeting in uppsala 23-24 february 2016 [powerpoint slides]. 2016.

62. Global Agricultural Information Network. Indonesia: Food and agricultural import regulations and standards - narrative. Fairs country report. Global Agricultural Information Network, 2015.

63. Iodine Global Network. Introducing salt reduction strategies without jeopardizing salt iodization: A who workshop in south-east asia. Iodine Global Network, 2015.

64. World Health Organization Regional Office for Eastern Mediterranean. Moving forward on salt and fat reduction in the region. Eastern Mediterranean Health Journal. 2015;21(5).

65. Rafieifar S, Pouraram H, Djazayery A, Siassi F, Abdollahi Z, Dorosty AR, Abtahi M, Kazemeini H, Farzadfar F. Strategies and opportunities ahead to reduce salt intake. Arch Iran Med. 2016;19(10):729-34. Epub 2016/10/16.

66. Hashemi S. Health promotion and holistic approach to nutrition in i. R. Iran [powerpoint slides]. 2016.

67. Zargaraan A, Dinarvand R, Hosseini H. Nutritional traffic light labeling and taxation on unhealthy food products in iran: Health policies to prevent non-communicable diseases. Iranian Red Crescent Medical Journal. 2017;In Press. doi: 10.5812/ircmj.57874.

68. Food Safety Authority of Ireland. Monitoring of sodium and potassium in processed foods. Period: September 2003 to december 2018. Ireland: Food Safety Authority of Ireland, 2019.

69. Safefood. Safetrak research 13 to 18 Ireland: Safefood; 2017 [cited 2019 15 December]. Available from: <https://www.safefood.eu/Publications/Market-research/Safetrak.aspx>.

70. Donfrancesco C, Strazzullo P, Galeone D, Palmieri L, Lo Noce C, Ippolito R, Russo O, Vanuzzo D, Giampaoli S. Trend of salt consumption in italy from 2009 to 2012. Circulation. 2018;129:AP406.

71. World Action on Salt and Health. Japan salt action summary 2016. Available from: <http://www.worldactiononsalt.com/worldaction/asia/japan/>.

72. National Institute of Health and Nutrition. Health japan 21 (the second term): National health and nutrition survey Japan: National Institute of Health and Nutrition; 2020 [cited 2020 03 January]. Available from: <https://www.nibiohn.go.jp/eiken/kenkounippon21/en/eiyouchousa/koumoku_eiyou_chousa.html>.

73. Al Jawaldeh A, Al-Khamaiseh M. Assessment of salt concentration in bread commonly consumed in the eastern mediterranean region. East Mediterr Health J. 2018;24(1):18-24. Epub 2018/04/17.

74. Kim C. Approaches to reducing population salt intake - republic of korea [powerpoint slides]. Korea Health Industry Development Institute, 2017.

75. Lina Jang. Government initiative to lower sodium intake pays off. The Korea Bizwire. 2016 19 December 2016.

76. World Health Organization. Kuwaitis lower blood pressure by reducing salt in bread: World Health Organization; 2014 [cited 2019 15 December]. Available from: <https://www.who.int/features/2014/kuwait-blood-pressure/en/>.

77. Almedawar MM, Nasreddine L, Olabi A, Hamade H, Awad E, Toufeili I, Arnaout S, Isma'eel HA. Sodium intake reduction efforts in lebanon. Cardiovasc Diagn Ther. 2015;5(3):178-85. Epub 2015/06/20. doi: 10.3978/j.issn.2223-3652.2015.04.09.

78. Taillie LS, Rivera JA, Popkin BM, Batis C. Do high vs. Low purchasers respond differently to a nonessential energy-dense food tax? Two-year evaluation of mexico's 8% nonessential food tax. Prev Med. 2017;105s:S37-s42. Epub 2017/07/22. doi: 10.1016/j.ypmed.2017.07.009.

79. Nieto C, Rincon-Gallardo Patiño S, Tolentino-Mayo L, Carriedo A, Barquera S. Characterization of breakfast cereals available in the mexican market: Sodium and sugar content. Nutrients. 2017;9(8). Epub 2017/08/17. doi: 10.3390/nu9080884.

80. Galina O, Elena R, Olga P. Informing national salt reduction strategy. The Moldovan Medical Journal. 2018;61(2):9-16. doi: 10.5281/zenodo.129901.

81. Republica Moldova. Hotărîre nr. 730 din 08.09.2014 u privire la aprobarea programului naţional în domeniul alimentaţiei şi nutriţiei pentru anii 2014-2020 şi planului de acţiuni pentru anii 2014-2016 privind implementarea programului naţional. 2014.

82. Enkhtungalag B, Batjargal J, Chimedsuren O, Tsogzolmaa B, Anderson CS, Webster J. Developing a national salt reduction strategy for mongolia. Cardiovasc Diagn Ther. 2015;5(3):229-37. Epub 2015/06/20. doi: 10.3978/j.issn.2223-3652.2015.04.11.

83. Derouiche A, Elkardi Y, Elarbaoui M, Elammari L, Azaz L, Alami M, Jafri A. Is the reduction of salt in the bread consumed in morocco could be a means of protection against cardiovascular diseases? Archives of Cardiovascular Diseases Supplements. 2020;12(1):157. doi: 10.1016/j.acvdsp.2019.09.324.

84. Al-Jawaldeh A, Rayner M, Julia C, Elmadfa I, Hammerich A, McColl K. Improving nutrition information in the eastern mediterranean region: Implementation of front-of-pack nutrition labelling. Nutrients. 2020;12(2). Epub 2020/01/30. doi: 10.3390/nu12020330.

85. Temme EHM, Hendriksen MAH, Milder IEJ, Toxopeus IB, Westenbrink S, Brants HAM, van der AD. Salt reductions in some foods in the netherlands: Monitoring of food composition and salt intake. Nutrients. 2017;9(7). Epub 2017/07/25. doi: 10.3390/nu9070791.

86. Heart Foundation. Heart foundation food reformulation targets. New Zealand: Heart Foundation, 2017.

87. Ni Mhurchu C, Eyles H, Choi YH. Effects of a voluntary front-of-pack nutrition labelling system on packaged food reformulation: The health star rating system in new zealand. Nutrients. 2017;9(8). Epub 2017/08/23. doi: 10.3390/nu9080918.

88. Ministry for Primary Industries. 2016 new zealand total diet study. Wellington 6140, New Zealand: Ministry for Primary Industries, 2018.

89. World Health Organization Regional Office for Europe. Meeting of the who action network on salt reduction in the population in the european region (esan): Meeting report 2-10 may 2017, dublin, ireland. World Health Organization Regional Office for Europe, 2017.

90. Directorate of Health. The salt partnership 2015–2018 - progress and goal achievement 0213 Oslo: Directorate of Health; 2019 [cited 2020 03 January]. Available from: <https://www.helsedirektoratet.no/rapporter/saltpartnerskapet-2015-2018-fremdrift-og-maloppnaelse>.

91. World Health Organization. Oman's experience on salt reduction in bread: World Health Organization; 2015 [cited 2019 15 December]. Available from: <https://www.who.int/beat-ncds/countries/oman/salt-reduction/en/>.

92. Hussein I, Al Ghannami S. Integrating salt reduction and salt iodization in oman. Iodine Global Network, 2019.

93. Ramlawi A, Rub AA, Materia E, Rossi L, Shuabi N, Barone M, Racalbuto V. Reducing dietary salt consumption in the occupied palestinian territory: A cross-sectional survey. The Lancet. 2018;391:S4. doi: <https://doi.org/10.1016/S0140-6736(18)30329-5>.

94. Ministerio de Salud. Resolucion n 049 (de martes 30 de enero de 2018). Panama: Gaceta Oficial; 2018.

95. Decreto supremo que aprueba el reglamento de la ley n° 30021, ley de promoción de la alimentación saludable: Dectero supremo n 017-2017-sa. El Peruano.

96. Michail N. Peru: Nutrition warning labels become mandatory: News and Analysis on Food and Beverage Development and Technology - Latin America; 2019. Available from: <https://www.foodnavigator-latam.com/Article/2019/06/17/Peru-Nutrition-warning-labels-become-mandatory>.

97. Portuguese Society of Hypertension. Salt forum: Expert panel recommendations. Lisbon, Portugal: Portuguese Society of Hypertension, 2015.

98. World Action on Salt and Health. Qatar 2017. Available from: <http://www.worldactiononsalt.com/worldaction/middle-east/qatar/>.

99. Vorotnikov E. Russia plots launch of traffic-light nutrition labels. Just-food. 2018.

100. Liverpool B. Implementation of svg multi-sectoral ncd action plan [powerpoint slides]. Saint Vincent and the Grenadines: Ministry of Health, Wellness and the Environment, 2016.

101. Trieu K, Ieremia M, Santos J, Neal B, Woodward M, Moodie M, Bell C, Snowdon W, Faumuina T, Webster J. Effects of a nationwide strategy to reduce salt intake in samoa. J Hypertens. 2018;36(1):188-98. Epub 2017/08/15. doi: 10.1097/hjh.0000000000001505.

102. Trieu K, Webster J, Jan S, Hope S, Naseri T, Ieremia M, Bell C, Snowdon W, Moodie M. Process evaluation of samoa’s national salt reduction strategy (masima): What interventions can be successfully replicated in lower-income countries? Implementation Science. 2018;13(1):107. doi: 10.1186/s13012-018-0802-1.

103. Health Promotion Board. Sample healthy workplace catering policy 2018. Available from: <https://www.hpb.gov.sg/workplace/workplace-programmes/useful-information-for-organisations/guidelines-and-tools/sample-healthy-workplace-catering-policy>.

104. Pravst I, Lavrisa Z, Kusar A, Miklavec K, Zmitek K. Changes in average sodium content of prepacked foods in slovenia during 2011-2015. Nutrients. 2017;9(9). Epub 2017/08/30. doi: 10.3390/nu9090952.

105. Department of Health. Foodstuffs, cosmetics and disinfectants act, 1972 (act 54 of 1972). Regulations relating to the reduction of sodium in certain foodstuffs and related matters: Amendment. Government Gazette; 2016.

106. Wentzel-Viljoen E, Steyn K, Lombard C, De Villiers A, Charlton K, Frielinghaus S, Crickmore C, Mungal-Singh V. Evaluation of a mass-media campaign to increase the awareness of the need to reduce discretionary salt use in the south african population. Nutrients. 2017;9(11). Epub 2017/11/16. doi: 10.3390/nu9111238.

107. The Heart and Stroke Foundation South Africa. Salt reduction in south africa: A report on the second high level salt reduction consultation meeting, south africa, 5 october 2018. Cape Town, South Africa: The Heart and Stroke Foundation South Africa, 2018.

108. de Lago M. Spain bans sale of unhealthy food in schools in bid to tackle obesity. 2011;342:d4073. doi: 10.1136/bmj.d4073 %J BMJ.

109. Bundesamt für Lebensmittelsicherheit und Veterinärwesen (BLV) [The Federal Food Safety and Veterinary Office]. Salzgehalt in schweizer broten konnte gesenkt werden [salinity in swiss bread could be reduced]: The Federal Food Safety and Veterinary Office; 2015 [cited 2019 15 December]. Available from: <https://www.admin.ch/gov/de/start/dokumentation/medienmitteilungen.msg-id-58497.html>.

110. Jaramillo J. Stakeholder and expert perspectives on dietary sodium reductions in thailand. World Health Organization, 2017.

111. Ministry of Public Health. Notification of ministry of public health (no. 374) b.E.2559 (2016). Re. Food products required to bear nutrition labelling and energy value, sugar, fat, sodium on the labels of some kinds of doods guideline daily amounts, gda labelling. 2016.

112. The World Bank. Using taxation to address noncommunicable diseases: Lessons from tonga. Washington, DC 20433, USA: The World Bank, 2019.

113. World Health Organization Regional Office for Europe. Progress in reducing salt consumption in turkey: World Health Organization Regional Office for Europe; 2013 [cited 15 December 2019]. Available from: <http://www.euro.who.int/en/countries/turkey/news/news/2013/04/progress-in-reducing-salt-consumption-in-turkey>.

114. World Health Organization Regional Office for Europe. Better noncommunicable disease outcomes: Challenges and opportunities for health systems. Country assessment: Turkmenistan. UN City, Marmorvej 51 DK-2100 Copenhagen Ø, Denmark: World Health Organization Regional Office for Europe, 2019.

115. World Action on Salt and Health. United arab emirates 2015 to 2017. Available from: <http://www.worldactiononsalt.com/worldaction/middle-east/united-arab-emirates/>.

116. Al Jawaldeh A, Rafii B, Nasreddine L. Salt intake reduction strategies in the eastern mediterranean region. East Mediterr Health J. 2019;24(12):1172-80. Epub 2019/02/26. doi: 10.26719/emhj.18.006.

117. MacGregor GA, He FJ, Pombo-Rodrigues S. Food and the responsibility deal: How the salt reduction strategy was derailed. 2015;350:h1936. doi: 10.1136/bmj.h1936 %J BMJ : British Medical Journal.

118. Public Health England. National diet and nutrition survey: Assessment of dietary sodium. Adults (19 to 64 years) in england, 2014. London SE1 8UG, UK: Public Health England, 2016.

119. Eyles H, Webster J, Jebb S, Capelin C, Neal B, Ni Mhurchu C. Impact of the uk voluntary sodium reduction targets on the sodium content of processed foods from 2006 to 2011: Analysis of household consumer panel data. Prev Med. 2013;57(5):555-60. Epub 2013/08/21. doi: 10.1016/j.ypmed.2013.07.024.

120. Wyness LA, Butriss JL, Stanner SA. Reducing the population's sodium intake: The uk food standards agency's salt reduction programme. Public Health Nutrition. 2012;15(2):254-61. Epub 2011/06/23. doi: 10.1017/S1368980011000966.

121. Public Health England. National diet and nutrition survey: Assessment of salt intake from urinary sodium in adults (aged 19 to 64 years) in england, 2018 to 2019. London SE1 8UG, UK: Public Health England, 2020.

122. U.S. Department of Agriculture, Agricultural Research Service. Nutrient intakes from food and beverages: Mean amounts consumed per individual, by gender and age, what we eat in america, nhanes 2011-2012. U.S. Department of Agriculture, Agricultural Research Service, 2014.

123. U.S. Department of Agriculture, Agricultural Research Service. Nutrient intakes from food and beverages: Mean amounts consumed per individual, by gender and age, what we eat in america, nhanes 2015-2016. U.S. Department of Agriculture, Agricultural Research Service, 2018.

124. Curtis CJ, Clapp J, Niederman SA, Ng SW, Angell SY. Us food industry progress during the national salt reduction initiative: 2009-2014. Am J Public Health. 2016;106(10):1815-9. Epub 2016/08/24. doi: 10.2105/ajph.2016.303397.

125. Odom EC, Whittick C, Tong X, John KA, Cogswell ME. Changes in consumer attitudes toward broad-based and environment-specific sodium policies-summerstyles 2012 and 2015. Nutrients. 2017;9(8). Epub 2017/08/05. doi: 10.3390/nu9080836.

126. John KA, Cogswell ME, Zhao L, Tong X, Odom EC, Ayala C, Merritt R. Change in us adult consumer knowledge, attitudes, and behaviors related to sodium intake and reduction: Summerstyles 2012 and 2015. Am J Health Promot. 2018;32(6):1357-64. Epub 2018/07/05. doi: 10.1177/0890117116679163.

127. World Health Organization Regional Office for Europe. Prevention and control of noncommunicable diseases in uzbekistan: The case for investment. UN City, Marmorvej 51 DK-2100 Copenhagen Ø, Denmark: World Health Organization Regional Office for Europe, 2018.

128. Do HT, Santos JA, Trieu K, Petersen K, Le MB, Lai DT, Bauman A, Webster J. Effectiveness of a communication for behavioral impact (combi) intervention to reduce salt intake in a vietnamese province based on estimations from spot urine samples. J Clin Hypertens (Greenwich). 2016;18(11):1135-42. Epub 2016/11/05. doi: 10.1111/jch.12884.

129. Social Republic of Viet Nam. National action plan on communication and advocacy for dietary salt intake reduction for prevention and control of hypertension, stroke and other non-communicable disease, period 2018-2025. Social Republic of Viet Nam, 2018.
